# Tuning Curves for Arm Posture Control in Motor Cortex Are Consistent with Random Connectivity

**DOI:** 10.1371/journal.pcbi.1004910

**Published:** 2016-05-25

**Authors:** Hagai Lalazar, L. F. Abbott, Eilon Vaadia

**Affiliations:** 1 Center for Theoretical Neuroscience, Columbia University, New York, New York, United States of America; 2 Department of Physiology and Cellular Biophysics, Columbia University, New York, New York, United States of America; 3 Edmond and Lily Safra Center for Brain Sciences, Hebrew University, Jerusalem, Israel; University College London, UNITED KINGDOM

## Abstract

Neuronal responses characterized by regular tuning curves are typically assumed to arise from structured synaptic connectivity. However, many responses exhibit both regular and irregular components. To address the relationship between tuning curve properties and underlying circuitry, we analyzed neuronal activity recorded from primary motor cortex (M1) of monkeys performing a 3D arm posture control task and compared the results with a neural network model. Posture control is well suited for examining M1 neuronal tuning because it avoids the dynamic complexity of time-varying movements. As a function of hand position, the neuronal responses have a linear component, as has previously been described, as well as heterogeneous and highly irregular nonlinearities. These nonlinear components involve high spatial frequencies and therefore do not support explicit encoding of movement parameters. Yet both the linear and nonlinear components contribute to the decoding of EMG of major muscles used in the task. Remarkably, despite the presence of a strong linear component, a feedforward neural network model with entirely random connectivity can replicate the data, including both the mean and distributions of the linear and nonlinear components as well as several other features of the neuronal responses. This result shows that smoothness provided by the regularity in the inputs to M1 can impose apparent structure on neural responses, in this case a strong linear (also known as cosine) tuning component, even in the absence of ordered synaptic connectivity.

## Author Summary

Relationships between the activity of single neurons and experimental parameters are often characterized by functions called tuning curves. Regular tuning-curve shapes are typically assumed to arise from structure in the synaptic inputs to each neuron. We found that the activities of neurons in primary motor cortex during an arm posture task exhibit both a regular component that fits a well-known tuning curve description, and heterogeneous irregular components that do not. Such complex components are often assumed to reflect residual noise. However, both the regular and irregular components are needed to optimally decode the commands that guide the muscles used in the task. We then asked what type of input structure was needed to generate neuronal responses with both regular and irregular elements. We constructed and analyzed a mathematical model, based on known physiology of the relevant brain regions that replicates the full spectrum of recorded neuronal responses. Surprisingly, the synaptic connectivity in this model is completely random.

## Introduction

The dependence of neuronal responses on stimulus- or movement-related parameters is often characterized by tuning curves. A natural assumption is that a smooth, regular tuning curve reflects structured, orderly input to a neuron. However, neuronal responses inevitably involve a degree of irregularity, even when responses are averaged across trials. Do such irregularities simply reflect noise in the inputs, or might they suggest something more complex such as unstructured input? Here we address this question using data recorded from primary motor cortex (M1) during an arm posture task, augmented by a neural network model of M1 neurons and their inputs.

Interpreting neural activity during arm movements is difficult because motor and sensory activity as well as limb biomechanical variables all change simultaneously. In this study, we focus on the often ignored task of actively maintaining arm posture. Arm posture control is a natural behavior without the dynamic complexity of time-varying movements. It is therefore well suited to address the nature of neuronal tuning curves and their relationship to input. To reveal fine-scale tuning curve structure, we employed a task with 54 different arm postures, consisting of 27 target positions with two forearm rotation angles.

Previous studies of arm posture control [1–3] concluded that neuronal responses in M1 vary as linear functions of hand position, which, by a change of coordinates, is equivalent to cosine tuning. The finding of broad, singly peaked tuning curves in both motor and visual areas led to proposals suggesting that both are generated by similarly structured cortical microcircuit [4, 5]. The M1 tuning curves we extract have a strong linear component, in agreement with previous studies, but we also find sizable and significant nonlinear elements. We examine the quality of the fit of linear tuning curve models across the entire population of tuned neurons, we analyze the nonlinear components in detail, and we determine how both linear and nonlinear elements contribute to the accuracy of inferring EMGs from M1 neuronal activity. Finally, we construct a neural network model of M1 activity driven by target-position related inputs that accurately replicates the data. Surprisingly, the synaptic connectivity in this model is completely unstructured, in fact, random. This model shows that the presence of a strong regular and, in this case, linear component in the tuning of a population of neurons does not necessarily imply structured connectivity.

## Results

**Fig 1:**
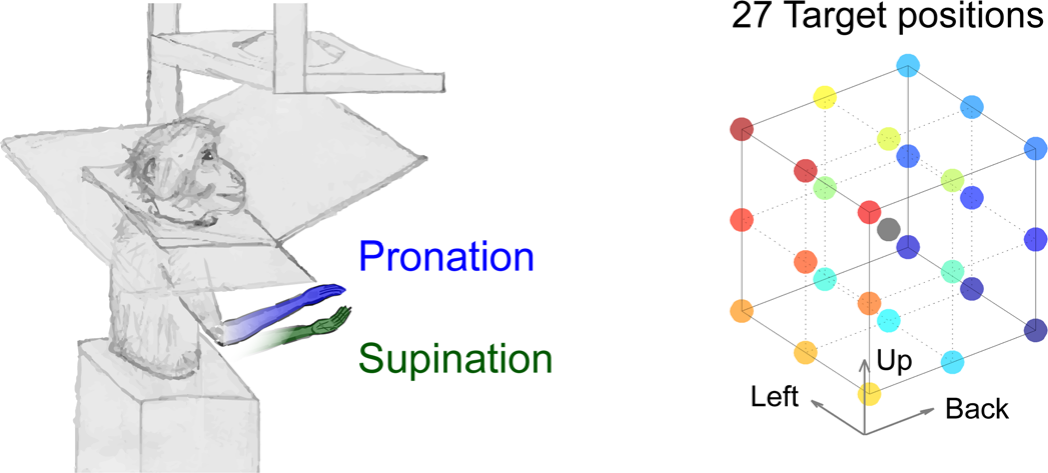
Monkeys controlled a cursor in a standard virtual-reality setup, where they held one of 27 targets in 3D space, with their forearm pronated or supinated.

During a continuous target-to-target reach and hold task in a virtual reality setup, monkeys maintained static arm posture at one of 27 targets in 3D space (Methods). They were trained to perform this task with their forearm at a pronated or supinated angle (Fig 1). To study posture control, we analyzed single-unit activity during a 200 ms period near the end of the target hold epoch when firing rates are approximately constant (S1B Fig). The responses of 81% (411/510) of the recorded neurons are tuned across the 54 arm postures (27 target positions times 2 forearm rotation angles; ANOVA, *p* < 0.01; statistical significance was also verified using the non-parametric Kruskal-Wallis test with identical results). Various tests established that arm-posture tuning did not arise from hand jitter (S1 Text).

### Tuning of the neuronal responses

**Fig 2:**
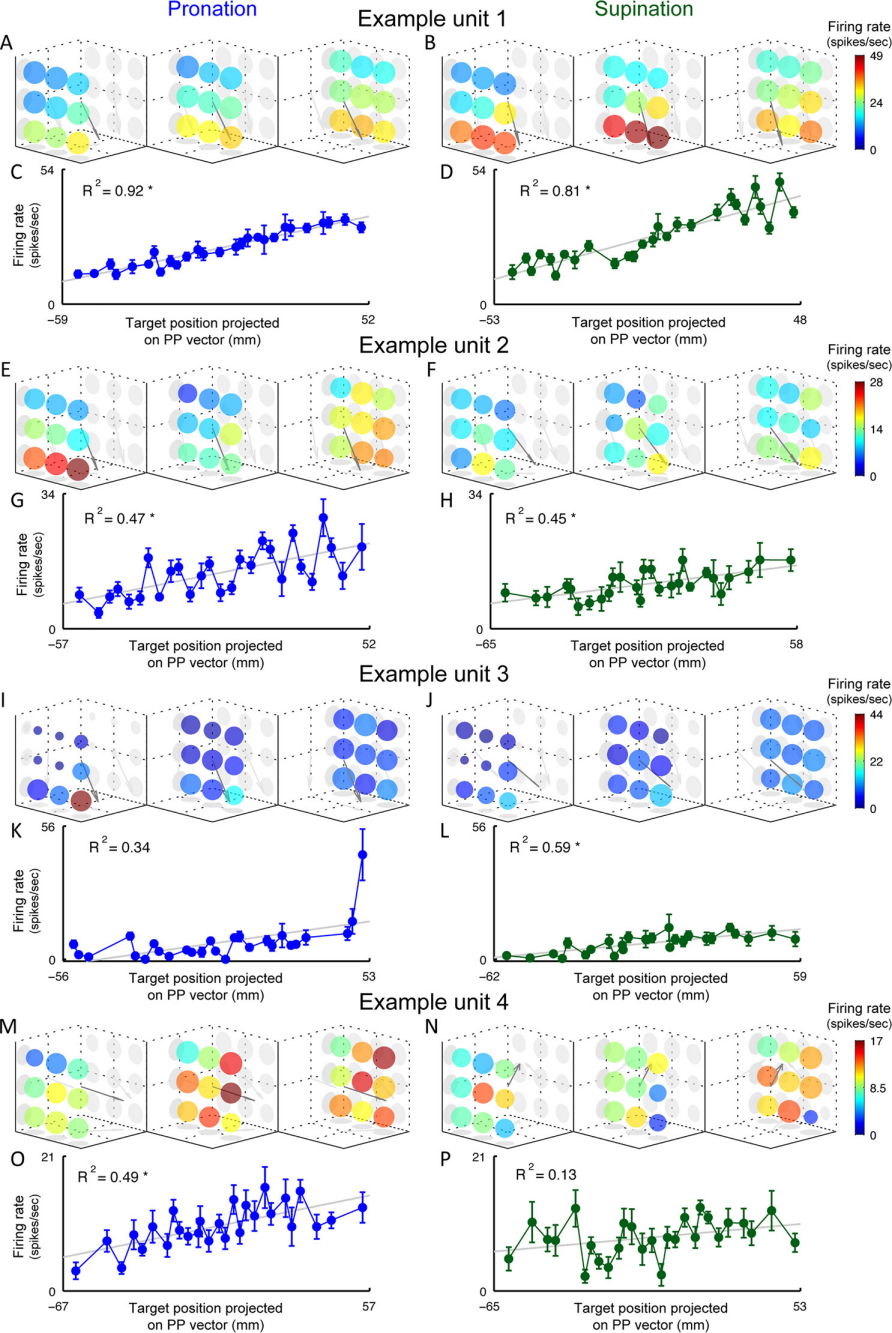
Examples of neuronal response functions. Colored plots show mean firing rates as function of 3D target position. Sphere radius is proportional to the confidence in the mean (SEM normalized by the mean), a smaller radius means lower confidence. The PP vector from the linear tuning-curve is plotted as an arrow. Left panel for pronation, right for supination. Below, mean firing rates ± SEM plotted as function of target positions projected onto PP vector. Gray line shows firing rates expected by linear tuning-curve, and its R^2^ (star denotes a significant F-test, *p* < 0.01). **A-D.** Neuron with one of the best fits to the linear tuning-curve in our dataset. Target positions perpendicular to the PP vector have almost equal firing rates. **E-H.** Neuron exhibiting spatial bimodality. **I-L.** Neuron with a spatially local response. **M-P.** Neuron with most common form of nonlinearity with high spatial frequencies (neighboring targets have large differences in firing rates).

**Fig 3:**
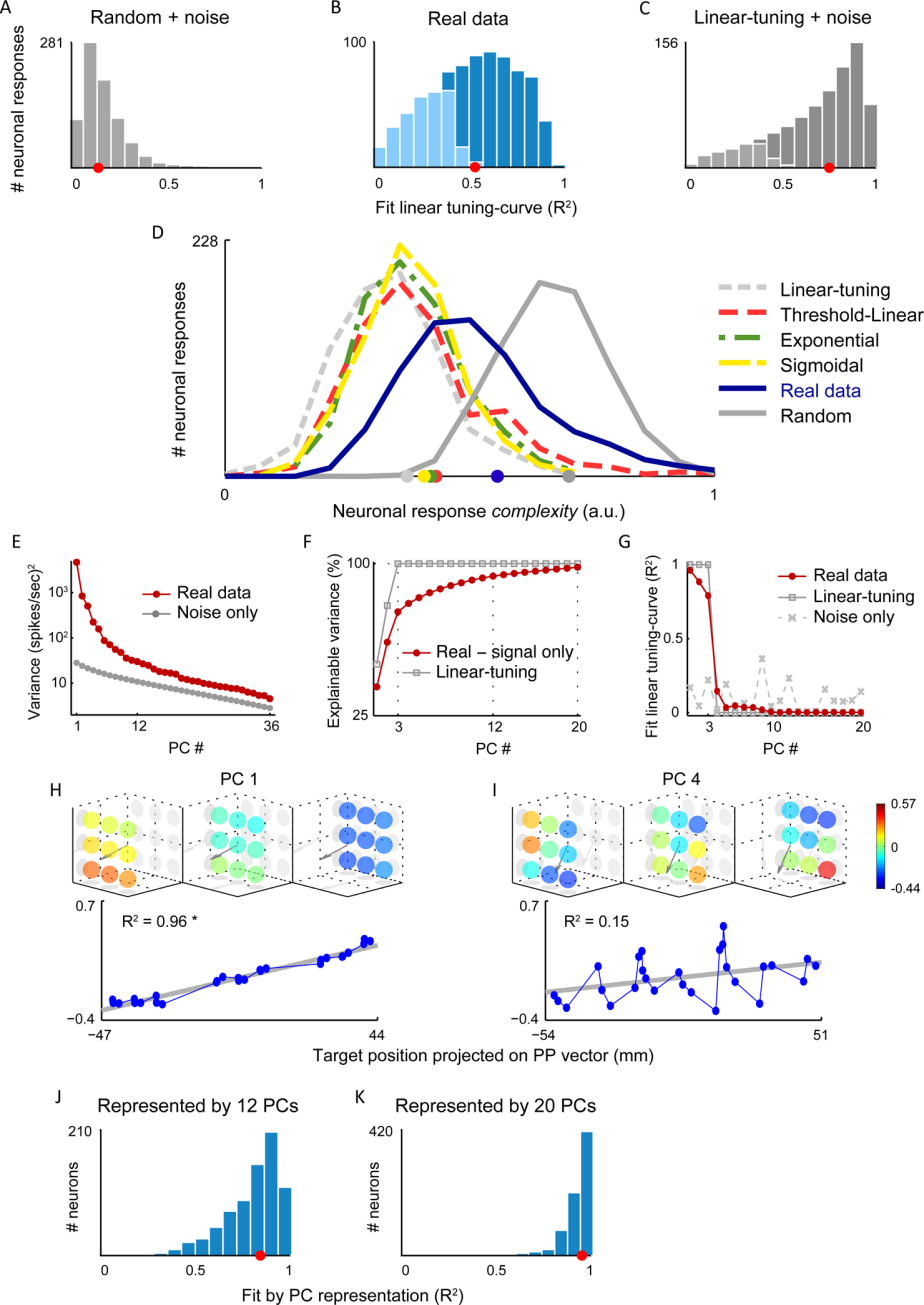
**A.** Distribution of R^2^ values for fit of linear tuning-curve to uniformly random artificial responses (median = 0.15), with addition of noise traces from real data (repeated 1,000 times). Almost all fits were not significant (F-Test, *p* < 0.01), denoted by light gray bars. **B.** Same as A but for real data, including the responses for all tuned neurons (*N* = 411) for both forearm rotation angles, significant fits (F-Test, *p* < 0.01) are denoted by dark blue (median = 0.52). **C.** Same for the artificial spatial linear control dataset. Distribution (median = 0.76) significantly different from that for real data (Wilcoxon rank-sum test, *p* < 10^−27^). **D.** Distribution of *complexity measure* across (normalized) response functions. Blue for the real neuronal responses, other colors for matched spatial linear, smooth nonlinear, and uniformly random control datasets, all with added real noise traces. Means (plotted on x-axis) significantly different for controls than for real data (*t*-test, *p* < 10^−40^ for the closest distribution). **E.** PC variances of the response functions across 54 conditions, and PC variances of noise traces sampled from the data (resampled 1,000). Variance explained by first 12 PCs of data is above variance of first noise PC, and variance of first 36 PCs of data is above the mean noise variance. The 99% confidence interval of the mean of the variance of the noise traces is indicated by the thickness of the gray line, and is smaller than the round markers. **F.** PCA of response function of both forearm angles (27 conditions each). Percent cumulative *explainable variance* as function of PC number, computed by subtracting the noise variance from total variance of the real data. In gray, the variance of purely spatial linear control (without noise). **G.** R^2^ values for fit of PCs to linear tuning-curve, for real data (red), purely spatial linear (gray and squares), or real noise traces (dashed gray and x’s). For real data and linear control only first 3 PCs have a significant fit to linear tuning-curve. Mean R^2^ for fit of each of noise PCs, across the 1,000 resamples is plotted, showing chance level for fitting noise to linear tuning-curve. **H.** 1^st^ PC vector of real data plotted in the same format as the neuronal responses (Fig 2A and 2C); here color denotes the PC coefficient. This PC vector is the most spatially linear and represents one dimension of the spatial linear component. **I.** Same for 4^th^ PC vector. This PC vector is highly nonlinear and is part of basis representing the nonlinear structure of responses. (See the first 20 PC vectors in S3 Fig). **J.** R^2^ distribution for the “model” formed by representing each response function by the first 12 PC vectors (median = 0.84). **K.** Same as H when using the first 20 PC vectors (median = 0.95).

The responses of each neuron, for a particular forearm angle, are summarized by a set of 27 mean firing rates, one for each hand position (Fig 2). To study the relationship between firing rate and hand position, we fit spatial linear tuning curves [2] to the responses of all the tuned neurons (N = 411; Methods), separately for pronation and supination. Neurons with perfectly spatial linear tuning would fire at rates that increase linearly when the hand is held at different positions along a *Preferred Position* (PP) vector and are constant across locations in planes orthogonal to this vector. During pronation, the response of the neuron shown in Fig 2A is well fit by a linear tuning curve (Fig 2C). However, this is one of only 9 neurons with R^2^ > 0.9 for the fit to linear tuning. Across the population of tuned neurons, the distribution of R^2^ values for the spatial linear tuning fit is broad (Fig 3B). We pooled the R^2^ distributions from both forearm angles because they are not significantly different (Wilcoxon rank-sum test, *p* = 0.56) and, in fact, there is only a small reduction in goodness of fit if the PP vectors for each neuron are required to be the same for both forearm angles (Methods, Eq 3). The R^2^ distribution has a median of 0.52, and its breadth indicates that neurons range from being well fit to poorly fit by a linear tuning curve.

We tested whether the deviations of the data from the linear fit are due to noise by generating artificial data with perfectly spatial linear tuning, using the model parameters obtained by fitting the real data, and including noise extracted from the real data (resampled 1,000 times; Methods). We calculated mean responses for these artificial data and re-fit them to the linear tuning curves. The resulting distribution of R^2^ values is skewed towards 1 (Fig 3C), with a median of 0.75, indicating significantly better fits than those for the real data (Wilcoxon rank-sum test, *p* < 10^−46^). Generating Poisson spike trains with rates given by the generated linear tuning curves over the same number of trials as the real neurons yielded similar results. We also studied whether the observed neuronal responses had linear structure above chance level by generating another artificial dataset constructed from randomly generated responses drawn across the same range of firing rates as the real neurons. Noise traces extracted from the data were added to these responses. When fit to the linear tuning curve, this resulted in significantly worse fits than for the real data, with an R^2^ distribution (Fig 3A) skewed towards zero with a median of 0.12. These analyses indicate the linear model is not a complete description of the tuning curves extracted from the data, but that the linear component is significantly larger than what would be expected from a random mapping between hand position and firing rate.

The neuronal responses deviate from spatial linearity in myriad ways. Some are bimodal, with high firing rates in two regions of space and low firing rates for the targets in between (Fig 2E). Other neurons have spatially localized tuning, responding to only one or two adjacent target locations (Fig 2I). Still others display more complex nonlinear spatial patterns, either on top of a linear component (Fig 2M) or without one (Fig 2N). Strikingly, most of the responses exhibit high spatial frequencies, with large differences in firing rates between neighboring targets in a seemingly unsystematic manner. This causes a zigzag pattern in the projected responses (Fig 2D, 2G, 2O and 2P). Such “salt and pepper” tuning does not support the explicit encoding of motor parameters (hand position, joint angles, etc.) by single neurons, because these should vary systematically with changes in hand position.

We quantified the spatial irregularity of each neuron’s responses using a *complexity measure*, based on the distribution of firing rate differences between all pairs of neighboring targets (Methods). The resulting distribution of spatial complexities across the real neurons (Fig 3D) covers values that are significantly larger than for linear and "regular" nonlinear (threshold-linear, exponential, sigmoidal; with added noise as described above) artificial datasets, yet significantly smaller than for an artificial dataset constructed from random responses. Stated another way, the real responses contain more power at high spatial frequencies than conventional parametric linear and nonlinear tuning curves, but not as much as expected for completely random tuning (Fig 3D).

Because the neuronal responses are not well described by standard parametric tuning curves (e.g. threshold-linear, exponential, sigmoidal, etc.), we used principal component analysis (PCA) to quantify their shapes non-parametrically. We also performed PCA on noise traces resampled from our data to implement a procedure developed by Machens et al. [6] for separating signal from noise (Methods). The first 12 PCs of the full data each account for more variance than the 1^st^ PC of the noise (Fig 3E; 99% confidence interval, bootstrap). Using this strict threshold, we find that the responses occupy 12 of the possible 54 dimensions, with the rest assumed to be noise. If, instead, we take the mean of the noise variance across all of its PCs as the noise threshold, the responses are 36 dimensional. These results do not depend on whether or not the data is normalized (Methods). We also performed PCA on the separate pronation and supination data sets, obtaining in each case 12 or 20 dimensions above the maximal or mean noise thresholds, respectively. Regardless of where in the range from 12–36 the exact dimensionality lies, it is much higher than the 3 dimensions expected for linear tuning, and it is presumably sufficiently high to allow these neurons to control the arm muscles required for this task.

To study the signal variance in the data, we subtracted away the noise variance and compared the resulting cumulative *explainable variance* to that of the artificial linear dataset, which is described by only 3 PCs (Fig 3F). The difference between these curves reveals the contribution of the heterogeneous nonlinear components. The first 3 PCs of the real responses roughly describe the linear component described above (F-Test, *p* < 0.01, Fig 3G, Fig 3H). The 4^th^ and higher PCs (Fig 3I) provide a basis for the heterogeneous nonlinear structure found in the responses (S3 Fig).

How much better can a general model describe the neuronal responses than the linear tuning curves? To answer this question we used the non-noise PCs to construct nonlinear “models” of the neuronal responses and compared these with the data. Using only 12 PCs, results in a dramatically improved description relative to the linear fit, with 93% of neuronal responses having an R^2^ > 0.52 (the median value for the linear tuning curve fit) and a median R^2^ of 0.84 (Fig 3J). Using 20 PCs produces an excellent fit for every neuron (median = 0.95; Fig 3K).

### Decoding EMG

**Fig 4:**
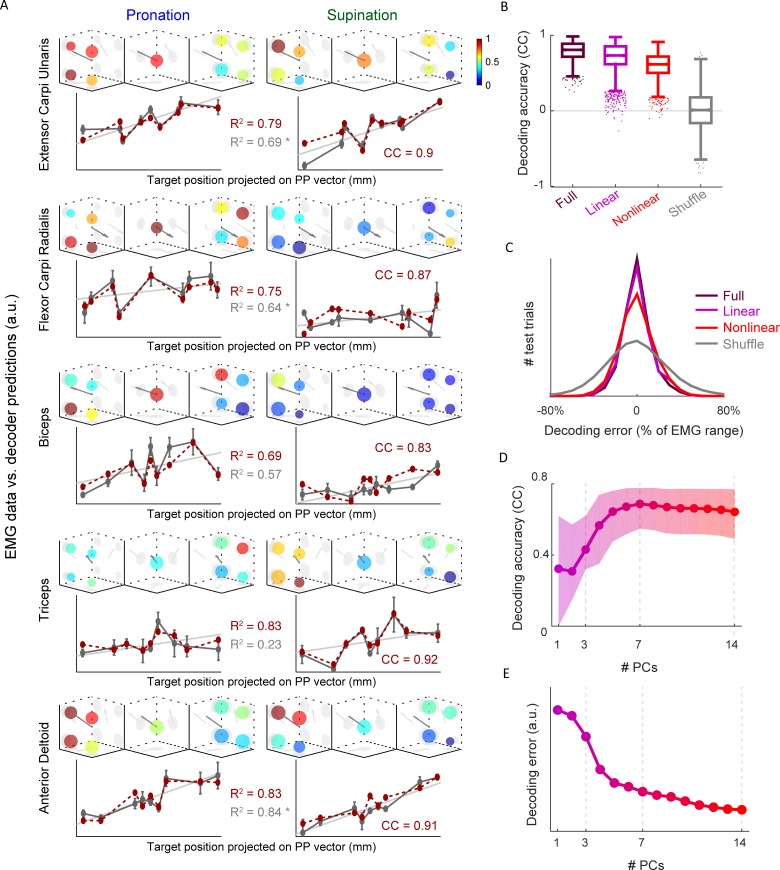
**A.** EMG as function of arm posture in same format as the neuronal responses (Fig 2). EMGs were fit to the extended linear tuning-curve (Methods, Eq 3), the PP vector in 3D plots, gray line in 2D plots, and R^2^ in gray, reflect this fit. Dotted red line shows EMG values predicted by linear decoder on held-out test data from one repetition of cross-validation, with its R^2^ and correlation coefficient in red. **B.** Distributions of decoding correlation coefficients between predicted and real EMG values for the test trials of each cross-validation, over all muscles. Box plot shows median, 25% and 75% percentiles, whiskers cover ~ 99% of distribution, dots are outliers. Decomposition of responses into linear and nonlinear datasets described in text (and S3D Fig). **C.** Distributions of decoding errors (real EMG minus predicted) for all test trials and all muscles. The Linear and Full datasets’ curves partially overlap. **D.** Decoding correlation coefficients, like in B., as a function of number of PCs that data is projected on to. PCs were learned on training data only, and normalized and trial-averaged before PCA. **E.** Same as D., for S.D. of decoding error.

To investigate the relationship between position-dependent tuning of M1 neurons and arm muscle activity, we studied how the EMG of the primary muscles used in the task could be decoded from M1 activity. To obtain sufficiently accurate measurements of EMG activity, we used the mean EMG (averaged over a time window, trials, and 6 days; Methods) recorded at 18 arm postures (9 targets times 2 forearm rotation angles; Fig 4A) rather than the full 27. Like the neuronal activities, the EMG of all 5 muscles had a significant nonlinear component, with 3 of the muscles significantly fit by a linear tuning curve as well (F-test, *p* < 0.01). To reconstruct these EMGs from M1 activity, we used single-trial neuronal firing rates combined from neurons recorded across different days (pseudo-simultaneous population activity; Methods). This is a reasonable strategy because the EMGs were averaged over trials and days as well. However we expect this to limit our ability to decode the EMG because using the mean EMG and pseudo-population activity breaks single-trial correlations.

We performed decoding using 1,000 different random cross-validation splits of the data into training and test sets (C.V. repetitions) each consisting of 18 test trials (one per arm posture). We obtained the best performance in cross-validation tests by using *optimal subset selection* [7] in which the linear decoder is constructed with a LASSO algorithm [8]. This selects the optimal subpopulation of neurons for decoding each muscle, rather than using all of the neurons to decode all of the EMGs (Methods). This has the additional benefit of providing information about how the signals that affect different muscles are distributed across the M1 population (S2 Text).

We first determined how well the EMGs could be decoded using the full neuronal responses (see Methods). The decoding predictions using the neural activity capture the nonlinear structure in the EMG even on a *single-trial* basis, outperforming the fit of the EMG to a linear tuning curve, which is based on the *mean* EMG values. The latter is the best that could be accomplished from the neurons if they had purely spatially linear responses and no noise. We assessed decoding performance using the full responses and also different response components separately, by dividing the data into distinct spatially linear and nonlinear components. To make this split, each single-trial firing rate was expressed as the sum of its underlying mean linear component (the value predicted by the linear tuning curve), the mean nonlinear component (the total mean firing rate minus the linear component), and a noise fluctuation for that trial (Methods; S4D Fig). We then constructed spatially linear and nonlinear datasets by keeping either the linear component and the noise or the nonlinear component and the noise, and we repeated our decoding procedure twice, using one or the other of these datasets.

Predicted EMG for the test trials of one C.V. repetition are compared with data for each muscle in Fig 4A. Using the full data, the median correlation coefficient between the decoded and actual EMG signals across the 1,000 C.V. repetitions is 0.81 (Fig 4B), and decoding errors are distributed around zero with a standard deviation of 13% of the min/max range of the EMG (Fig 4C). The decoding accuracy using only the linear component (median CC = 0.73, S.D. decoding error = 14%; Fig 4B and 4C) is somewhat worse than decoding using the full data, and decoding using only the nonlinear components is slightly worse still (median CC = 0.62, S.D. decoding error = 16%; Fig 4B and 4C). Chance level, as quantified by a shuffle control, resulted in a median correlation coefficient of 0.01, and decoding errors with a standard deviation of 29% (Fig 4B and 4C). Thus, the EMG could be decoded far above chance level using either the linear or nonlinear datasets, and the nonlinear component contributed to the accuracy of the full decoding.

In a second approach, we decoded the EMG using increasingly complex nonlinear datasets by projecting the data onto increasing numbers of PCs of the neuronal responses (as described previously). As PCs representing nonlinear structure are added, the decoding accuracy improves, achieving the maximal (median) correlation coefficient with 7 PCs (Fig 4D) and the minimal (standard deviation) decoding error with 14 PCs (Fig 4E). These results confirm that nonlinear tuning contributed significantly to the information about muscle activity contained in the recorded M1 responses.

### Non-separable interaction between hand position and forearm angle

We now address the impact of forearm angle on hand-position tuning, starting by looking for two simple effects, an additive shift and a multiplicative gain change between the two forearm angles. To examine a possible linear shift, we computed the baseline firing rate for each neuron, defined as the lowest firing rate across targets. The distribution of baseline firing rate differences (S.D. = 4.3 spike/s) was significantly greater than what would be expected purely from noise (bootstrap control; S.D. = 1.4 spikes/s; F-test for variances, *p* < 10^−48^), indicating a significant additive shift between the firing rates for the two forearm angles. To look for gain changes, we examined the range of the firing rates across all targets for the two forearm rotation angles. Again, the distribution of gain differences (S.D. = 8.7 spikes/s) was significantly broader than for the bootstrap control (S.D. = 3.3 spikes/s, F-test for variances, *p* < 10^−31^), indicating a gain change.

**Fig 5:**
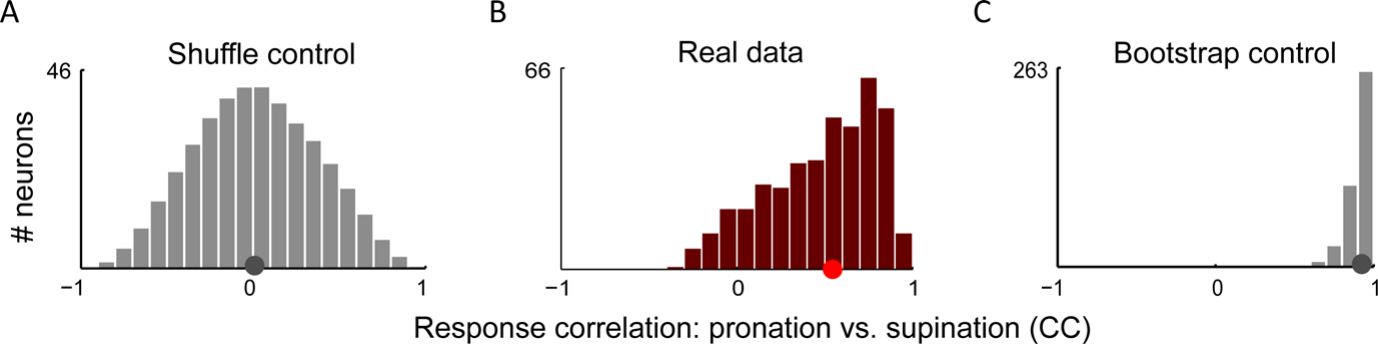
**A.** Distribution of correlation coefficients between pronation and supination for shuffle control, created by computing correlation coefficient between each pronation response function and supination response of 1,000 other neurons (with replacement), median = 0. **B.** Same for responses of real (tuned) neurons, median = 0.54. **C.** Same, for a bootstrap control, median = 0.93, drastically different than for real data (Bootstrap of the medians, *p* < 10^−64^).

If baseline shifts and gain changes were the only impact of forearm rotation, the correlation coefficient between the firing rates of any neuron across the two forearm angles would differ from 1 only due to noise effects (Methods). Instead, we found that the distribution of correlation coefficients between responses for pronation, and supination is broad (Fig 5B, median = 0.54) and very different from the distribution expected solely from noise (Fig 5C, median = 0.93, bootstrap of the medians, *p* < 10^−64^). On the other hand, most of these correlation coefficients are positive, and their distribution is not at all like that for correlation coefficients between random pairs of responses (Fig 5A). Thus, the *shapes* of the hand-position tuning curves change as a function of forearm angle beyond a baseline shift and gain change, but not as much as if the tunings in these two cases were unrelated. This suggests that the independent task variables (the joint angles constrained by target position, and the forearm rotation angle) are *non-separable* at the level of M1, and thus they appear to have undergone nonlinear mixing.

### A random feedforward model replicates the data

In order to further understand the data, we searched for a minimalistic neural network model with biologically realistic constraints that could give rise to the empirical results. The nonlinear component of the responses we have studied appears quite random, but the presence of a strong linear component would seem, at first sight, to rule out a model based purely on random target-related inputs to M1 neurons. There is, however, a complication because physiological constraints impose a degree of smoothness on the tuning curves, and a random set of responses might exhibit a degree of regularity, including linearity, purely because of smoothness. To address the degree to which smoothness constraints impose a linear component on responses to random inputs, we constructed a model in which M1 neurons are driven by inputs conveying information about target location.

**Fig 6:**
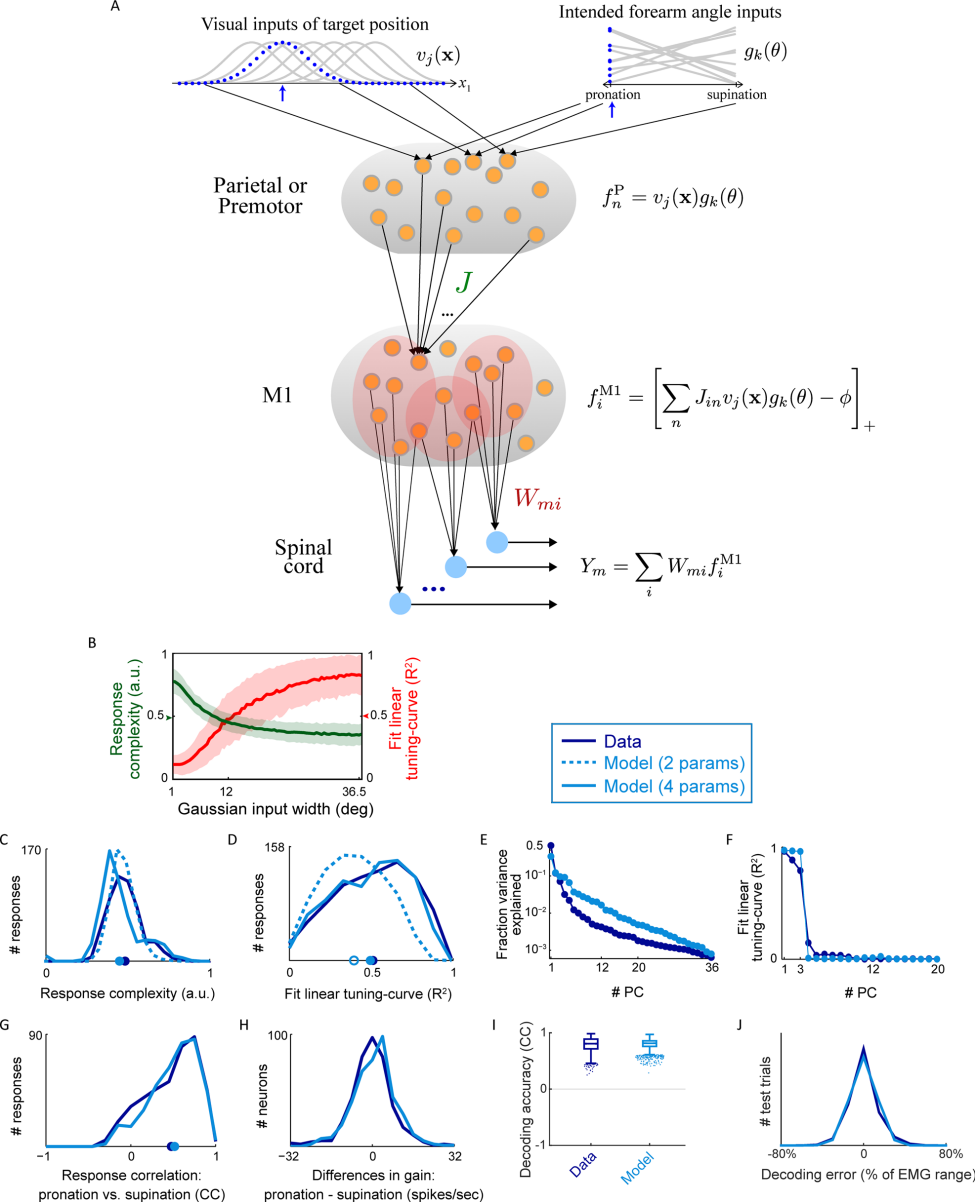
**A.** Feedforward neural network model for arm posture control. Visual inputs conveying target position are represented by array of 3D Gaussian units (here illustrated in 1D). For given target position (blue arrow) population response is a Gaussian bump (blue dots). Inputs representing intended forearm rotation angle are carried by second population of input neurons, each having a random preference for either pronation or supination, the population of responses for a pronation condition is shown in blue. The shape of their tuning curves for intermediate angles were not sampled in our task; they are drawn as lines only for illustration. Presynaptic parietal or premotor responses are modeled as a multiplicative interaction of the two input streams from a subset of “nearby” inputs from each (to maintain tuning). Each M1 response function receives a random set of 10,000 presynaptic inputs, but only a small fraction of these are active at any target. These are mixed through random connectivity *J* and passed through a threshold-linear nonlinearity. The EMG for each muscle is obtained from a linear combination of a subpopulation of such M1 neurons. These synaptic weights are the only ones in the model that require adjustment. **B.** Mean ± S.D. of response function complexity (left vertical axis) and R^2^ of fit to the spatial linear tuning-curve (right vertical axis) of model-generated responses (2-parameter model), as a function of the Gaussian width parameter for the visual inputs. This single parameter accounts for the higher spatial-linearity (wider Gaussian inputs) or higher spatial complexity (narrower Gaussian inputs) found in different response functions in the population. Arrows mark the mean values for the real data. **C.** Distribution of response function complexity for real data (blue, re-plotted from Fig 3D, mean = 0.49), and for the model-generated responses for the 2-parameter model (teal dashed line, mean = 0.49) and for 4-parameter model (solid line, mean = 0.46). Although only the mean of the data distribution was used for fitting, the model reproduces the entire shape of the data distribution well. **D.** Distributions of R^2^ values for fits to the linear tuning-curve for real data (mean = 0.5, S.D. = 0.22; re-plotted from Fig 3B), 2-parameter model (mean = 0.4, open circle, S.D. = 0.19), and 4-parameter model (mean = 0.49, S.D. = 0.23). **E.** Fraction of total variance explained of the PCA of the (54 conditions) real (adapted from Fig 3E) and model generated response functions. **F.** R^2^ values for fits of PCs to linear tuning-curve for the data- (Fig 3G) and the model-generated responses. **G.** Distribution of correlation coefficients between the pronation and supination responses for each neuron for the data (Fig 5B; mean = 0.48, S.D. = 0.31) and the model (mean = 0.51, S.D. = 0.28). This result involves no additional parameter fitting. **H.** Distributions of differences in gain for each neuron across forearm angles for the data (S.D. = 8.7 spikes/s) and the model (S.D. = 8.4 spikes/s). We scaled response of each model neuron for both forearm angles to match the range of firing rates of a randomly chosen real neuron without using any forearm angle information in this scaling. **I.** EMG decoding correlation coefficients using the real data (Fig 4B) and the model response functions. **J.** Same for the distributions of decoding errors (Fig 4C).

In the model, target position is represented by a population of neurons that have Gaussian receptive fields (one dimension of which is illustrated in Fig 6A) centered on particular 3-dimensional preferred target locations. This is consistent with the properties of parietal reach area neurons [9–11], but this population could also correspond to neurons in premotor cortex. The model input neurons have receptive fields with different preferred target locations and, at first, they have the same widths. Later, we extend the model to include more realistic heterogeneity in input receptive field widths.

Each target generates a Gaussian “bump” of activity across the population of input neurons. Each M1 neuron is connected to a random selection of 10% of the input neurons. As a result, each M1 neuron received about 330 inputs that are active somewhere in the workspace and about 50 active inputs at each target location. These inputs are multiplied by random synaptic weights (Methods) and the result is summed to produce the total input for each neuron. The response of each model M1 neuron is then computed by passing its total input through a threshold-linear firing-rate function. The threshold for this response function depends on a threshold parameter that, in this initial model, is set to the same value for all M1 neurons to match the mean *coding level* of the real neurons (0.85). The coding level is the fraction of conditions that cause a neuron to respond either at a level significantly different from 0 (*p* < 0.01, *t*-test, Bonferonni corrected) or ≥ 5 spikes/s.

Once the coding level is set, this first-round model has only a single free parameter, the input tuning curve width (our model fits are not sensitive to other features of the model, such as the total number of input-layer neurons, and the number and strength of synapses per M1 neuron). This width determines the smoothness of the M1 responses and this, in turn, controls the tradeoff between spatial-linearity and complexity (Fig 6B). Wider input receptive fields lead to increased smoothness and spatial linearity, whereas narrower fields increase spatial complexity. For a receptive field width corresponding to a visual angle of 12°, the model M1 responses have the same mean complexity measure as the real neurons (Fig 3D). Interestingly, this width is consistent with the values reported for parietal cortex neurons [9, 12].

Surprisingly, this simple random model does a good job of matching both the linear and nonlinear components of the real neurons, (S5 Fig, compare to Fig 2). Even though we only used the mean complexity and mean coding level of the data to set the 2 parameters of the model (the input tuning-curve width and the M1 threshold), the entire complexity measure distribution for the model responses is very similar to that of the real neurons (Fig 6C). In addition, the distribution of R^2^ values for the fit to the linear tuning curve is similarly broad (Fig 6D). Thus a feedforward model receiving input with structured tuning through random synapses can account for both the spatially linear and nonlinear components without any explicit tuning of its synaptic connections (i.e. with purely random connectivity).

The 15% undershoot in the standard deviation of the linear tuning curve R^2^ distribution (Fig 6D) for the two-parameter model can be improved by extending the model slightly. In the extended model, each M1 neuron is assigned a threshold to match the coding level of one of the real neurons (randomly chosen), and the input receptive field widths are drawn independently from a uniform distribution parameterized by a mean and range (Methods). We chose the mean and range of the tuning curve width distribution to obtain the best match between the model and data for the mean and standard deviation of the linear tuning curve R^2^ distribution (Fig 6D).

The neuronal responses of the enhanced model match the real data extremely well (Fig 6C–6J). The fraction of variance explained by the PCs of the model data matches the general shape seen for the real data, although the nonlinear components of the model are represented slightly more uniformly across its PCs (Fig 6E). This shows that the model responses capture approximately the same dimensionality as the real data. In addition, the first 3 PCs of the model neurons capture the spatial linear component as they do for the real data (Fig 6F). We also checked whether the M1 model could reproduce the recorded EMG activity. To do this, we added a set of linear readouts to the model (Fig 6A, bottom) and adjusted the weights of these readouts to fit the EMGs, using the decoding scheme used for the real neurons (described above). Relative to the small number of muscles, the neurons form an overcomplete basis. As a result, using the noise-free model neurons decodes the EMGs almost perfectly. For a fair comparison to the performance of the real data, we added noise samples to the model neurons, resampled from the real data (Methods). Decoding the EMGs using the model neurons with noise produced decoding correlation coefficients (median CC = 0.82 for the model vs. 0.81 for the data; Fig 6I) and decoding errors (S.D. = 12% of EMG range vs. 13%, respectively; Fig 6J) comparable to that of the real neurons. These decoding results provide additional confirmation that the model M1 neurons capture the dimensionality and frequency content of the real neurons.

To model the effects of forearm rotation angle, we let the input neurons that drive the model M1 neurons depend on forearm angle as well as on target location (Fig 6A, top right). The forearm angle dependence involves multiplying the Gaussian function of target location for each neuron by a factor, chosen independently for each input neuron, that takes a different value for pronation or supination, with the values chosen randomly (tuning of the forearm factor could have any shape for intermediate values, since these were not sampled in our task). Thus, the forearm angle inputs act as a gain modulation of the visual inputs consistent with responses found in premotor cortex [13] and eye-position gain field modulation of visual responses in parietal cortex [12, 14]. With forearm angle included in this way, the correlations between the model neuronal responses for pronation and supination are extremely similar to those for the real neurons (98% similar in the mean and S.D. of the two distributions; Fig 6G), without any additional parameters or fitting. The distributions of differences in gain across forearm angles are also very similar (S.D. for the data = 8.7 versus for the model = 8.4 spikes/sec, Fig 6H). These results are a consequence of the random nonlinear interaction of visual target and forearm angle signals at the input stage of the model, suggesting a mechanism that may underlie the non-separable mixing in M1 discussed above.

## Discussion

Cosine tuning has played a dominant role in discussion of the tuning of M1 responses during reaching movements [15–18] but, due to many conflicting findings, this approach has fallen out of vogue [19–23]. During arm posture control, we found that M1 neuronal responses show a heterogeneity of irregular nonlinear responses that includes high spatial frequencies (Fig 2), in addition to the linear (cosine-tuned) component described previously [1–3]. Due to this complex nonlinear component, it is unlikely that any reasonable coordinate choice could lead to a simple parametric description of these responses. This is in contrast to the long-standing tradition of using parametric tuning curves and encoding models to describe neuronal responses. Despite its irregularity, the nonlinear response component contributes to decoding the EMG of the major muscles used during arm posture control (Fig 4).

It is natural to assume that regular neuronal responses imply structured (and in network models, learned) input synaptic connectivity [24–26]. However, we found that random connectivity could reproduce the data (Fig 6C-6J), with regularity, in particular linearity, arising from smoothness. This smoothness stems from assumptions about the nature of the inputs to M1, assumptions that are consistent with the known physiology of the target-position coding regions [9, 12]. Importantly, this smoothness was not imposed by limits on the resolution provided by the task (as an extreme example, a task with only two targets would unavoidably produce linear tuning) or by the analyses we performed [27, 28]. Despite its simplicity and small number of adjustable parameters, the model accounts for not only the linear (Fig 2A) and nonlinear (Fig 3D) components, but also their distributions across the neuronal population (Fig 6). This is a rare example in which both the regularity *and* the diversity of a population of neural responses have been replicated by a neural network model. More generally, the model provides support for the idea that some neural circuits use random connectivity to generate rich and high-dimensional representations, and produce their outputs by tuning only their output synaptic weights [29–32].

Firing rates were constant during posture control and feedback did not have a measurable effect on the neuronal responses (S1 Text). This implies that the population has either relaxed into fixed points or is dominated by the (target position and intended forearm angle) inputs external to M1. Either way, any internal recurrent inputs from other M1 neurons cannot generate the tuning to arm posture, which is why we focused on a feedforward network architecture. Recurrent dynamics are likely to play an important role in how M1 generates time-varying movements, such as reaching [33, 34]. Such recurrent connections are unlikely to be random and may require synaptic modification [35].

A number of influential theoretical models of M1 function have taken a normative engineering-inspired approach [36–40]. By combining the equations of motion of a model arm with putative population M1 responses with features such as cosine tuning, these models can account for many psychophysical features of arm movements. The normative approach has helped clarify the relationships between the arm’s biomechanics and resulting movement variables, and has highlighted the caution necessary in interpreting the correlations between movement variables and neuronal responses. However, as has long been acknowledged [36], due to the considerable dimensionality reduction from M1 to muscles, biomechanical properties do not generally suffice to constrain models of cortical computation. The model we have presented here does not attempt to be a general model of M1 function, but it does incorporate representations in the inputs to M1 that are consistent with known physiology and neuronal properties that are biologically realistic. We believe that understanding M1 computation will require models that integrate *neural* constraints, in addition to constraints imposed by the periphery.

The idea that sensory inputs are mapped to motor commands through a series of sequential coordinate transformations has received a lot of attention [41–45]. In this view, sensory representations lead to kinematic representations and only finally to joint torques and muscle activity. However, many daily actions require combinations of task demands, involving hand position, arm posture, endpoint and segmental forces. Therefore, intended endpoint forces, for example, must sometimes be represented by the inputs to M1 and not only by its outputs. In general, the sensorimotor system must be able to process parallel sensory and intentional signals, each ultimately shaping the activity of the same muscles. Our experiment involved demands on both hand position and forearm rotation angle, a degree of freedom that does not affect hand position. The neural responses show an interaction between these two independent inputs that is non-separable (i.e. cannot be decomposed into a sum or product of functions; Fig 5). This suggests that these signals are already mixed nonlinearly before the level of M1. Most encoding models introduced in the M1 literature, whether linear [46–48] or nonlinear [49, 50], produce separable functions. In this view, M1 responses are an example of *nonlinear mixed-selectivity* [32], representations that have been shown to have considerable computational power in tasks requiring nonlinear combinations of multiple parameters [51]. We incorporated this finding into our model by basing the inputs to the model M1 neurons on the multimodal visual and arm-posture-dependent receptive fields found in premotor cortex [13, 52–56] and posterior parietal cortex [57–62]. The model places the idea that such multimodal activity reflects the multiplexing of several sensorimotor parameters [63, 64] within the context of a class of basis-function network models [65–67]. In this view, the multimodal responses found in parietal and premotor cortex may be evidence for the nonlinear mixing of parallel task variables, in addition to sequential sensorimotor transformations. Because of this basic feature of its construction, our model predicts that during experiments with additional concurrent task demands, M1 responses should show non-separable interactions across all task dimensions. This can be tested experimentally, by, for example, repeating our experiment with two different load conditions at the hand, in addition to varying hand position, and forearm rotation.

Posture control could, in principal, be implemented by a simple “on-off” cortical signal that causes ongoing spinal feedback to maintain current muscle activation levels. Even a transiently tuned cortical motor command could activate ongoing spinal control [68]. In contrast to these possibilities, we found that most M1 neurons are tuned even 1–1.8 seconds after a target is reached and posture is held constant. By using a powerful decoding algorithm, some of the hand jitter during the target hold epoch could be predicted [69]. However, having found no correlations between firing rates and hand jitter suggests that such signals are weak and that the ongoing M1 responses primarily represents a feedforward (intentional) motor command, with ongoing corrections executed by sub-cortical feedback loops. This is in contrast to the strong and fast feedback corrections seen in M1 in response to large unexpected perturbations of the arm [70].

The muscle activations required for a motor task are typically nonlinear functions of task parameters. In principle, M1 neurons could encode task parameters linearly and only downstream spinal cord circuits would generate the required nonlinearities. Alternatively, the required nonlinearities may already be present in M1 responses, which could then be read out in a purely linear manner by spinal cord circuits (an idea we incorporated into our model; Fig 6A). Obviously, these are two extremes, and the truth probably lies between them. Nevertheless, our data show that the nonlinearities needed for correct muscle activation are already present in the M1 responses, and these can be read out linearly to produce the recorded EMGs (Fig 4). In a series of studies, Miller and colleagues found an invariant relationship between M1 and EMG activity by decoding EMGs in a variety of reaching and grasping tasks [71–73]. In our study, by first studying the statistical structure of the population of response functions (Fig 3) and then combining analyses of encoding with decoding (Fig 4B–4E), we showed that the full structure of the M1 responses, both the spatial-linear component and heterogeneous nonlinear components, contribute to creating the EMG. This diversity provides a basis with high-dimensionality (Fig 3E), providing enough dimensions in neural space to span the space of independently controllable muscles of the arm. By using the subset selection algorithm, we found that specific subpopulations of M1 neurons more optimally control individual muscles, or groups of muscles. These subpopulations partially overlap (S4A–S4C Fig), consistent with the gross topography found in M1 [74].

Neural representations involve a compromise between two incompatible demands: accuracy and noise tolerance. M1 arm-posture related responses, situated between the noise tolerant properties of regular (linear) tuning curves and the high degree of accuracy provided by irregular tuning, provide an example of such a compromise. An interesting prediction of the model, related to this observation, involves the increased motor accuracy associated with foveating a target. The presentation of a visual target initiates a saccade that brings it to the fovea [75], improving target localization [76]. By what mechanism does the resulting increased in visual acuity benefit motor execution? The dependence of M1 response complexity on the width of the target-position inputs in our model (Fig 6B) suggests an answer. Because foveal receptive fields are narrower [10], fixating the target should bias the smoothness-randomness tradeoff of M1 neurons towards randomness, increasing response complexity with higher spatial frequencies and resulting in more accurate motor commands. This prediction can be tested experimentally by comparing M1 neurons while arm posture is maintained at a visual target viewed peripherally versus foveally.

## Methods

Two monkeys (Macaca fascicularis; PK—4.3 kg male, and BR—3.4 kg female) were trained in our behavioral task, in a standard 3D virtual reality setup (Fig 1). They controlled a cursor by active LED markers attached to their right hand, measured by a motion capture system (Phoenix Technologies, sampled at 100 Hz), while their left arm was comfortably restrained. An opaque panel concealed their arms from view.

Animal care was in accordance with the National Institutes of Health guidelines and was approved and supervised by the Hebrew University committee on animal experiments. The animals were housed in groups of four in an open playroom that included trees, toys, and enrichment. Surgery involved the use of standard anesthetics (Kamacaine, Medetomidine, Ketamine, and Isoflurane), antibiotics (Cefazoline), and analgesics (Carprofen) that were administered pre-, during, and post-operatively as needed. Throughout the experiments, the animals were carefully monitored 7 days a week (i.e. including rest days), and after conclusion of the experiments were retired to a primate shelter park.

### Neural & EMG recording

After training, a chronic 96 microelectrodes array with 1.5 mm electrodes (Blackrock Microsystems) was implanted in the arm area of contralateral rostral M1 (S1A Fig) using the surgery protocol described elsewhere [77]. Single-unit action potentials were sampled at 30 kHz and sorted using an automatic adaptive spike-sorting algorithm with human adjustment (Blackrock Microsystems). Spike waveforms were inspected visually offline and, due to the large neuronal yield, only very well isolated single units were included. A firing-rate stability analysis was performed in a semi-supervised manner using a Hidden-Markov Model that segmented even subtle changes in firing rate. Only neurons with firing rate that was stable throughout the recording session and had at least 1 spike/minute were included in the analysis. Therefore our inclusion criteria was based only on recording quality, and introduced no task specific selection bias. We included 378 neurons from monkey PK, 132 neurons from monkey BR. After verifying that the dataset from each monkey produces equivalent results (S2 Fig), we report all 510 neurons together.

Electromyography (EMG) was recorded on 6 days from monkey PK, from the Anterior Deltoid, Biceps, Triceps, Flexor Carpi Radialis, Extensor Carpi Ulnaris, using double-differential surface electrodes with pre-amplifiers x 20 (Motion Lab Systems). The raw EMG signals were down-sampled, rectified, and root-mean squared (20 ms window), and normalized per muscle for each day.

### Behavioral task

Monkeys made continuous, instructed-delay, target-to-target reaching movements between 27 targets in 3D space. On each trial, after reaching the target and receiving a drop of food reward (275 ms later), the monkeys had to maintain the static position of their hand at the target and their current forearm angle for another 750–1,500 ms (*target hold epoch*). After successfully reaching the target, any deviation from the target would cause the trial to fail and the screen to go blank for 1–2 seconds. This target hold epoch was before any of the sensory cues of the upcoming trial, including the next target, had appeared. The monkeys were trained to perform the task with their forearm in either the pronated (palm-down) or supinated (palm-up) postures (Fig 1), and these were instructed in alternating blocks of 25 trials. The cursor’s graphics were the same in both forearm angles, and in this way we dissociated the kinematics of the cursor from the biomechanical state of the arm. The 27 targets were arranged in a virtual cube (3 x 3 x 3), where the distance between the 2 furthest targets was 12.1 cm. This distance was a significant portion of the full range of motion for these monkeys, as the lengths of their upper limbs were 23.3, 21 cm from shoulder to wrist for monkeys PK and BR, respectively. Target radii were 1.7 cm for monkey PK (and 2 cm on some control days), and 2.5 cm for monkey BR. Data was analyzed from 38 recording days for monkey PK and 10 recording days for monkey BR. On nine control days of monkey PK’s experiment and when recording the EMG, only 9 targets (the corners and center of the virtual cube) were presented.

### Data analysis

We analyzed the data during a 200 ms period in the target hold epoch, which was 1,025–1,775 ms after reaching the target (750–1,500 ms after the reward), and 50 ms before the first instruction of the next trial (S1B Fig). The time ranges are due to an experimental design with randomly variable intervals. Both firing rates and hand position were most stable during this period. Firing rates were calculated by averaging over this 200 ms time window and mean firing rates over trial repetitions to the same *condition* (target position and forearm rotation angle). There was a mean of 14 trial repetitions, per condition, per day, giving a sizeable sample to estimate the underlying firing rates.

Spatial linear tuning. To measure the linear component of the spatial structure of each neuronal response, we fit them to the linear tuning curve described previously [2] using multiple linear regression:

$${\bar{r}}^{linear}\left(x\right)=a_{0}+a_{1}x_{1}+a_{2}x_{2}+a_{3}x_{3}$$ (1)

where the mean firing rate of each neuron, ${\bar{r}}^{linear}\left(x\right)$, at the mean hand position (binned by targets) **x** = (*x*_1_, *x*_2_, *x*_3_) is a linear function of the position, with regression coefficients *a*_0_, *a*_1_, *a*_2_, *a*_3_. If we define the *Preferred Position Vector* as **PP** ≡ (*a*_1_, *a*_2_, *a*_3_), we can write this equation equivalently in polar coordinates as:

$${\bar{r}}^{linear}\left(x\right)=a_{0}+\|\mathrm{PP}\|\cdot \|x\|\cdot \text{cos}\;\varphi_{\mathrm{PP},x}$$ (2)

where *φ*_**PP**,**x**_ is the angle (in radians) between the mean hand position and the **PP** vector. The **PP** vector points in the direction in space along which the firing rate increases linearly, and its norm, ||**PP**||, is the slope of this linear modulation. According to this model, the firing rates at hand positions in each plane orthogonal to this vector should be equal.

To quantify the linear dependence of the firing rates on the full arm posture (i.e. forearm rotation angle in addition to hand position), we additionally fit each neuron to two other equations. An *Extended* spatial linear tuning curve fits all 54 conditions with one **PP** vector but allows different baseline firing rates for each of the two forearm angles,

$${\bar{r}}^{extended}\left(x\right)=a_{0}+\|\mathrm{PP}\|\cdot \|x\|\cdot \text{cos}\;\varphi_{\mathrm{PP},x}+a_{4}\theta_{\text{f}}$$ (3)

where *θ*_f_ is the angle of forearm rotation. A third equation accounts for the forearm rotation angle exerting a multiplicative, instead of an additive, effect,

$${\bar{r}}^{multiplicative}\left(x\right)=a_{0}+g(\theta_{\text{f}})\cdot \|\mathrm{PP}\|\cdot \|x\|\cdot \text{cos}\;\varphi_{\mathrm{PP},x}$$ (4)

where ***g*** is defined to be 1 when ***θ***_**f**_ **= *p*** and a value determined by fitting when ***θ***_**f**_ **= *s***, for pronation and supination, respectively.

Extracting and quantifying hand jitter. We detected hand jitter by finding instantaneous hand speeds that exceeded the median of the distribution of maximal jitter speeds per trial (2.48 cm/s), and then found the beginning of each hand jitter using an algorithm developed by Amos Arieli [78]. Each trial could have between zero to several jitters. Different choices of the threshold, which slightly changed the number of jitters included in this analysis, had no significant effect on the results. Mean 3D directions were calculated from the hand jitter segments using orthogonal regression and were distributed in all directions in 3D space. Using multiple linear regression, we fit the instantaneous firing rates to the velocity using the multiplicative velocity tuning curve [48]:

$$r\left(t-\tau \right)=b_{0}+b_{1}\cdot \|\dot{x}\|+b_{2}\cdot \|\dot{x}\|\cdot {\mathrm{PD}}^{\prime }\cdot x$$ (5)

where the mean firing rate, *r*, with a lead/lag of *τ* ms to the beginning of the hand jitter, is a linear function of the hand’s speed, $\left\|\dot{x}\right\|$, and direction of movement, **x** (in Cartesian units), and ^***‘***^ denotes transpose. **PD** is the *preferred direction*—the normalized vector of the direction-specific regression coefficients—and *b*_2_ is its norm. For each neuron we calculated the firing rates in 100 ms windows, repeating the fitting procedure from neuronal activity leading behavior by 300 ms, to lagging behavior by 100 ms, in 10 ms increments. This was done for each forearm angle separately, and we report the maximal R^2^ for each neuron across all temporal lags. We repeated this analysis with either the full firing rates or the firing rate fluctuations (i.e. minus the mean rate for that condition).

Principal component analysis of the neuronal responses. Principal component analysis (PCA) [79] was applied to the responses of the significantly tuned neurons for the full 54 conditions response functions, and then again for each of the forearm rotation angles separately (27 conditions). We calculated PCA for 376 neurons, after excluding 35 tuned neurons from control days with only 9 targets so as not to introduce a bias at the targets they were not sampled.

We used the method described by Machens [6] to calculate an upper bound on the variance of the residual noise by calculating the PCA for *noise trace* estimates sampled directly from the data. Briefly, let the single-trial firing rate of the *i*-th neuron in the *c*-th condition for the *k*-th trial be denoted *r*_*i*_(*k*, *c*). After averaging over the trials for each condition, its response function is ${\bar{r}}_{i}\left(c\right)$, where the bar denotes the average over trials. The residual noise estimates were created by sampling two different single-trial firing rates randomly for each condition, subtracting them, and normalizing:

$${\bar{\eta }}_{i}\left(c\right)=\frac{1}{\sqrt{2K}}\left(r_{i}(k,c)-r_{i}(l,c)\right)\;,\;k\neq l$$ (6)

where there are *K* trials for this condition, and the bar denotes that this is an estimate of the average noise trace (over trials) for that neuron and condition. Subtracting two single-trials removes the shared underlying signal component, leaving a noise fluctuation that is from the same noise distribution. ${\bar{\eta }}_{i}\left(c\right)$ can be conceived as the residual noise *trace* remaining from averaging over a finite number of trials. Clearly as $K\to \infty ,\;{\bar{\eta }}_{i}\left(c\right)\to 0$. We then calculated the PCA separately for this dataset of noise traces, which produces an upper bound on the variance explained by the noise in the data. For the complete derivation of this method see the supplementary materials of [6]. We repeated this procedure 1,000 times (each repetition choosing a pair of single trials *k* ≠ *l* for each neuron and each condition) to calculate bootstrap estimates of the 99% confidence intervals for the variance of the noise.

To control for the potentially disproportionate effect of neurons with large firing rate ranges, we repeated the analysis by first normalizing each response function (mean subtraction and normalizing their range to 1). Only significantly tuned neurons were used for the PCA analysis to avoid magnifying neuronal noise by normalization. To control for neurons with high firing-rate variance, alternatively, we pre-processed the single-trial firing rates with the variance-stabilizing transformation for the Poisson distribution [80], which replaces *r*_*i*_(*k*, *c*) with its square root $\sqrt{r_{i}(k,c)}$. The un-normalized data and both of these normalizations produced essentially the same results.

Complexity measure. In order to measure the complexity of each neuron’s response function non-parametrically, we calculated the discrete derivative (Lipschitz continuity) between the mean firing-rate at each target, $\bar{r}\left(x_{k}\right)$, and at each of its closest targets, $\bar{r}\left(x_{l}\right)$. We defined the *complexity measure* for that *i*-th response function, as the standard deviation over these values:

$$\text{complexity}\left(i\right)=\text{stdev}\left\{\frac{\left|\bar{r}\left(x_{k}\right)-\bar{r}\left(x_{l}\right)\right|}{\sqrt{\sum {\left(x_{k}-x_{l}\right)}^{2}}}|\forall \;k,l,\text{s}.\text{t}.\sqrt{\sum {\left(x_{k}-x_{l}\right)}^{2}}=\;D_{min}\right\}$$ (7)

where, *k* and *l* are pairs of targets with the minimal distance *D*_*min*_. We used the normalized responses (with range 1, described above), in order to compare neurons with different firing rates. We compared the distribution of this complexity measure across the real neuronal responses to a purely spatial-linear control dataset (firing rates generated from the fitted linear tuning curve parameters, of each neuron) and several nonlinear controls, which passed the spatial-linear response through an additional nonlinearity (threshold-linear, exponential, or sigmoidal). We matched the range of firing rates of these controls to those of the real neurons and added noise traces (as described above). The complexity measure was calculated for 1,000 resamples of the noise traces, and the mean distribution for each control was reported.

Joint angle tuning curves. The five arm joint angles relevant to this task, shoulder abduction/adduction, shoulder flexion/extension, shoulder rotation, elbow flexion/rotation and forearm rotation, (both monkeys kept their wrist in a stereotyped posture throughout the task) can be divided into 4 that are determined by hand position, which we denote by the vector ***θ***(**x**), and the forearm rotation angle ***θ***_**f**_. While the monkeys did not have a single stereotyped elbow swivel angle across the entire workspace, elbow swivel was consistent as a function of the target position held, and hence is considered a member of ***θ***(**x**).

We first consider tuning curves in which the average firing rates can be expressed as

$$\bar{r}\left(x,\;\theta_{\text{f}}\right)=h\left(\theta \left(x\right)\right)+g\left(\theta_{\text{f}}\right)$$ (8)

where *h* and *g* are arbitrary functions. The difference between the response functions (based on hand position) across the 2 forearm angles is

$$\bar{r}\left(x,\;\theta_{\text{f}}=p\right)-\bar{r}\left(x,\;\theta_{\text{f}}=s\right)=g\left(\theta_{\text{f}}=p\right)-g\left(\theta_{\text{f}}=s\right)\equiv C$$

where *p* and *s* stand for pronation and supination, and *C* is constant that does not depend on hand position. The correlation coefficient between each neuron’s pair of responses should only deviate from 1, due to noise because

$$\text{corr}\left(\bar{r}\left(x,\;\theta_{\text{f}}=s\right),\;\bar{r}\left(x,\;\theta_{\text{f}}=s\right)+C\right)=1$$ (9)

If instead we assume a multiplicative interaction for the encoding of the forearm rotation with the other joint angles,

$$\bar{r}\left(x,\;\theta_{\text{f}}\right)=h\left(\theta \left(x\right)\right)\cdot g\left(\theta_{\text{f}}\right)$$ (10)

where *h* and *g* are again arbitrary. In this case, the response functions (based on hand position) will be scaled by a gain as a function of forearm angle,

$$\bar{r}\left(x,\;\theta_{\text{f}}=p\right)-\bar{r}\left(x,\;\theta_{\text{f}}=s\right)=h\left(\theta \left(x\right)\right)\cdot \left(g\left(\theta_{\text{f}}=p\right)-g\left(\theta_{\text{f}}=s\right)\right)$$

and again there is no change in response function shape,

$$\text{corr}\left(h(\theta (x))\cdot g(\theta_{\text{f}}=p),\;h(\theta (x))\cdot g(\theta_{\text{f}}=s)\right)=1$$ (11)

These equations simply make explicit the repercussions of the fact that forearm rotation is independent of hand position and therefore independent of the joint angles that are constrained by it. The extended and multiplicative spatial linear tuning curves (Eqs 3 and 4) are non-parametric versions of each of these types of models (Eqs 8 and 10, respectively).

Decoding EMG. Using single-trial neural activity, we decoded the mean EMG of the five muscles we recorded (Fig 4A). The EMG was decoded in 18 conditions (9 targets per forearm rotation angle) by using the neural activity of *N* = 131 neurons that had at least 15 repetitions for each of these specific conditions. Pseudo-simultaneous population activity was created by concatenating randomly sampled single-trials from each neuron from the same condition. That is, to create one vector of pseudo-simultaneous population activity for the condition *c*, each neuron contributed a single-trial firing rate of one trial from that condition. Some of those firing rates may have been recorded simultaneously by chance, but on average most were not, effectively averaging out any single-trial (“noise”) correlations. This is a reasonable choice because we decoded *mean* EMG that averaged out single-trial correlations too. Decoding was carried out in a cross-validated scheme: for each repetition (“fold”) of cross-validation, 14/15^th^ of the data (*T* = 252 trials) were randomly chosen for training and the remaining 1/15^th^ (*T* = 18 trials) for testing. This was done in a balanced design, such that each test dataset included exactly one repetition for each condition. Cross-validation was repeated 1,000 times.

The linear decoder is an *N* x 5 matrix **W** of weights such that

Y=R′⋅W (12)

where **R** is the *N* x *T* matrix with each column equal to the population activity for one pseudo-simultaneous trial, **R’** is its transpose, **Y** is the *T* x 5 matrix of the mean EMG activity of the 5 muscles. We initially estimated the linear decoders using the *Moore-Penrose Pseudoinverse* (since our case is under-determined):

W=pinv(R′)⋅Y (13)

An approach that uses all the neurons for decoding can produce suboptimal performance on cross-validated data. We achieved improved decoding performance by using the LASSO algorithm [8], which selects an optimal subset of neurons. This learning algorithm is a regularized version of least squares estimation, that minimizes the decoder’s *L*_1_-norm in addition to the sum of squared residuals, leaving a smaller number of regressors with nonzero weights. The LASSO estimate is found by solving the following (quadratic programming) optimization problem:

$$W={\text{argmin}}_{W}\left\{\frac{1}{2}\sum_{t=1}^{T}{\left(y_{t}-\sum_{i=1}^{N}r_{ti}W_{i}\right)}^{2}+\lambda \sum_{i=1}^{N}\left|W_{i}\right|\right\}$$ (14)

This algorithm was repeated for the EMG of each muscle separately, allowing a distinct optimal subpopulation for each muscle. *y*_*t*_ is the EMG activity of the given muscle on trial *t*, *r*_*ti*_ is the firing rate of *i*-th neuron on that trial, and *W* is now a weight vector. The meta-parameter λ weights the relative importance of decoding accuracy (left term) versus the regularization (right term) and was selected as the value that minimized the mean squared error on a 5-fold cross-validation of the training data alone. The neural data was mean-centered, where the means were learned only from the training data and likewise for the intercept.

Decoding performance was quantified by the distribution of prediction errors: real EMG minus predicted value, for each of the 18 test trials, each repetition of cross-validation, and for each muscle. In addition, we computed the (Pearson) correlation coefficient between the series of EMG values for the 18 test trials and their respective prediction values. The distribution of correlation coefficients is over the repetitions of cross-validation and muscles.

Decomposition of single-trials into linear vs. nonlinear components. To compare the decoding performance of the nonlinear and the linear components separately, we decomposed the neural activity into 2 distinct datasets. Each single-trial firing rate, ***r***_***i***_(***k***, ***c***), for the *i*-th neuron on the *k*-th trial in condition *c*, was decomposed into a sum of its underlying mean spatial-linear component (the value predicted by the tuning curve at that hand position), ${\bar{r}}_{i}^{\mathrm{linear}}(c)$, the mean nonlinear component (the total mean firing rate minus the spatial-linear component), ${\bar{r}}_{i}^{\mathrm{nonlinear}}(c)$, and that trial’s noise fluctuation, ***η***_***i***_(***k***, ***c***), as defined above:

$$r_{i}\left(k,c\right)={\bar{r}}_{i}^{linear}\left(c\right)+{\bar{r}}_{i}^{nonlinear}\left(c\right)+\eta_{i}\left(k,c\right)$$

This is illustrated in S4D Fig for one example neuron’s response. To create the dataset comprised of purely spatial-linear components plus single-trial noise, we subtracted from each single-trial firing rate the mean nonlinear component for that condition (S4D Fig, bottom panel)

$${\bar{r}}_{i}^{linear}\left(c\right)+\eta_{i}\left(k,c\right)=r_{i}\left(k,c\right)-{\bar{r}}_{i}^{nonlinear}\left(c\right)$$ (15)

This is identical to taking the spatial-linear tuning curve’s predicted value for that condition and adding the trial’s noise. Equivalently, subtracting from each single-trial firing rate its respective spatial-linear component, creates a dataset of purely nonlinear components plus single-trial noise (S4D Fig, top panel)

$${\bar{r}}_{i}^{nonlinear}\left(c\right)+\eta_{i}\left(k,c\right)=r_{i}\left(k,c\right)-{\bar{r}}_{i}^{linear}\left(c\right)$$ (16)

This is identical to taking the residuals (from the fit to the spatial-linear tuning curve) and adding that trial’s noise fluctuation. We repeated the decoding procedure (described above) twice, using each of these datasets as inputs separately. Our decomposition relies on identifying the responses with a significant fit to the spatial-linear component, we therefore repeated this analysis with either *p* < 0.01 or *p* < 0.05 thresholds.

Feedforward neural network model. To discover what are minimal biologically realistic constraints that could give rise to our data, we built and analyzed a feedforward neural network model (Fig 6A). We assumed that visual information about target position is represented by an array of 3D Gaussian input units, consistent with the known receptive field properties of parietal reach area neurons [9–11]. The response of the *j*-th visual input unit at target position **x** is given by

$$v_{j}\left(x\right)=a_{0}\cdot \text{exp}\;\left(-\frac{1}{2}\left(x-\mu_{j}\right)\prime \cdot \Sigma_{j}^{-1}\cdot \left(x-\mu_{j}\right)\right)$$ (17)

where **μ**_*j*_ is the center of this unit’s receptive field, *a*_0_ is a normalization that was set such that the population response at each target would sum to 1, and Σ_*j*_ is a diagonal matrix of variances, $\sigma_{j}^{2}$. While in reality there may be neurons with non-spherical receptive fields, we started with this assumption to see what minimal structure is necessary to account for the diversity of M1 responses. In such an array target position is encoded by neuron identity, and each target results in a population response that is also a Gaussian bump. Since visual acuity is much higher than motor precision, we simulated 1,000,000 visual input units (which tiled space 100 x 100 x 100) to avoid any discretization artifacts. We had their centers, **μ**_*j*_, extend 3 times past the furthest target positions used in our task (in both directions and along each spatial dimension), to avoid boundary effects.

Intended forearm rotation angles *θ* were modeled as an additional set of input units, *g*_*k*_(*θ*), each randomly preferring pronation or supination and each with a uniformly random slope, such that for a pronation preferring neuron for example

$$g_{k}\left(\theta \right)∼\{\;\begin{array}{l} U\left[1,2\right],\;\text{if}\;\theta =\text{pronation}\\ U\left[0,1\right],\;\text{if}\;\theta =\text{supination} \end{array}$$ (18)

where *U* denotes a uniform distribution over the indicated range. The distributions are reversed for a supination preferring neuron. Since we only sampled forearm rotation at two points, we did not make any assumptions about these neurons’ tuning for intermediate values of *θ* (although we depicted them graphically as lines in Fig 6A). Alternatively, we modeled intended forearm rotation angle inputs by a 1D array of Gaussians as well, which produced similar results. The presynaptic inputs into M1, $f_{n}^{P}$, were modeled as (premotor or parietal) neurons that multiplicatively mixed the two input streams.

$$f_{n}^{P}\left(x,\theta \right)=v_{j}\left(x\right)g_{k}\left(\theta \right)$$ (19)

This construction assures that the neurons exhibit the tuning that depends on both visual targets and arm posture found in many premotor [13, 52–56], and posterior parietal areas [57–62].

Finally, our model M1 neurons were modeled as each receiving a random combination of 10,000 such inputs mixed through random connectivity, and passed through a threshold-linear nonlinearity

$$f_{i}^{M1}\left(x,\theta \right)={\left[\sum_{n}J_{in}f_{n}^{P}\left(x,\theta \right)-\phi_{i}\right]}_{+}$$ (20)

where *J*_*in*_ is the random connection between the *n*-th presynaptic neuron and the *i*-th M1 neuron, *ϕ*_*i*_, is its threshold, and the square brackets denote the threshold-linear function. Each element *J*_*in*_ is chosen independently and randomly from a uniform distribution between 0 and 1. Because the model tries to capture tuning curve shape and not absolute firing rates, only the shape of the distribution of the random connections described by *J* matters, not their magnitude.

In the first model we used (Fig 6C and 6D, S5 Fig), the input tuning widths and threshold were all set the same values, so the model depends on only 2 parameters. The threshold *ϕ* was set to insure that the coding level of the model matched that of the real neurons (0.85). The coding level is defined as the fraction of conditions that causes a neuron to respond, which we took as being either significantly different than 0 spikes/s (*p* < 0.01, *t*-test, Bonferonni corrected), or at least 5 spikes/s. The visual Gaussian width *σ* was chosen (visual angle of 12°) as to produce response functions with a mean complexity measure that fit the value for the real data.

In the enhanced version of our model (Fig 6C–6J), the threshold for each model neuron was chosen independently so that its responses matched the coding level of a randomly assigned real neuron. In addition, the visual tuning-curve widths were chosen randomly independently for each input neuron from a uniform distribution with a mean *σ*_*M*_ with a range *σ*_*R*_

$$\sigma \;∼\;U\left[\sigma_{M}-\frac{\sigma_{R}}{2},\;\sigma_{M}+\frac{\sigma_{R}}{2}\right]$$ (21)

*σ*_*M*_ and *σ*_*R*_ were fit by grid search (*σ*_*M*_ ∈ [20,120], *σ*_*R*_ ∈ [0,200]) and nonlinear optimization over the multi-objective optimization problem of trying to match the real neurons’ mean complexity measure and the mean (0.5) and standard deviation (0.22) of their R^2^ distribution for the linear tuning curve. There is a continuous range of values for which this version of the model is near optimal, and we simply chose one set of values for producing Fig 6C–6J.

In order to use the model neurons to decode the EMG, we followed the same decoding scheme used for the real neuronal responses, as described above. Since the model generates mean responses, to generate single-trials we added noise as follows. First, we created *noise samples* by subtracting randomly chosen pairs of single-trial firing rates, for each condition, from each real neuron:

$$\eta_{i}\left(c\right)=r_{i}\left(k,c\right)-r_{i}\left(l,c\right)\;,k\neq l$$ (22)

This is similar to the noise traces defined above but without the normalization (Eq 6). These noise samples were resampled from the data to match the number of trials per condition of the real dataset. Next, we created a lookup table of these noise samples across all the real neurons, where each row contains all the noise traces associated with the original mean firing rate, ${\bar{r}}_{i}\left(c\right)$, from which it was generated. Finally, single-trials firing rates were generated for each model neuron by adding noise samples extracted from the lookup table, by choosing the firing rate closest to the model neuron’s mean firing rate, at that condition. We chose to assign noise samples from the real to model neurons by using the mean rates as the lookup in order to capture the signal dependence of neuronal variability.

## Supporting Information

S1 Fig **A.** Chronic electrode array after insertion (for monkey BR; same location was used for monkey PK). C.S. = Central sulcus, A.S. = Arcuate sulcus. **B.** Mean ± S.D. of hand speed during the reaching movements to the target and the target-hold epochs, for each forearm posture. The 200 ms window analyzed in this study is denoted in red. Time is relative to task events; MB = Movement Beginning, and FC = Forearm rotation angle Cue (first sensory cue of next trial). Wide gray bar overlays the period where trials had random length intervals. Note that the hand speed is extremely low during target hold, yet not identically zero, because the hand was held freely in space. The larger variance before movement beginning is during the reaction time, and the larger variance during movement is due to averaging over reaches of different amplitudes. **C.** Mean ± S.D. of maximal hand jitter speed per trial, as a function of target position, and for each forearm posture. The hand jitter was uniform across targets (Multiple comparisons, using ANOVA, *p* > 0.01). **D.** Distribution of R^2^ values for fitting single trial firing rate fluctuations to hand jitter using the velocity tuning-curve, median = 0.12. Values with a significant fit (F-Test, *p* < 0.01) are in dark gray, while the rest are in light gray. Fitting to all tuned neurons (*N* = 411) and the optimal R^2^ for each neuron across all leads/lags (of firing rate to behavior) and forearm angles is presented. **E.** Same as D for control that shuffled single-trial firing rate fluctuations relative to their respective jitter movements, median = 0.11.
(TIF)

S2 Fig **A.** Distributions of R^2^ values for fit of linear tuning-curve for all tuned neurons (same as Fig 3B), separately from each monkey (means = 0.54 and 0.5, for monkeys BR and PK, respectively). **B.** Distributions of *complexity measure* of the (normalized) response functions (same as Fig 3D), separately for each monkey (means = 0.5 and 0.49, for monkeys BR and PK, respectively).
(TIF)

S3 Fig The first 20 PCs of the 54-condition PCA (Fig 3E), in the format of the neuronal responses (Fig 2). (TIF)

S4 Fig **A.** Percent of neurons selected for decoding each muscle, with distributions over cross-validation repetitions. Medians = 49%, 43%, 43%, 48%, 44%, for the forearm extensor, flexor, biceps, triceps, and deltoid, respectively (grand total median = 44%). **B.** Same as A., for percent of neurons selected for decoding any single muscle, any pair of muscles, etc., in each cross-validation repetition. Medians = 30% selected for any single, 27% for any pair, 24% for triples, 15% for quadruples, and 5% of neurons selected for decoding all 5 muscles. **C.** Matrix of mean correlations between decoders for each pair of muscles (only significant correlation coefficients were used, *p* < 0.01, Bonferroni corrected), averaged over cross-validation repetitions. This matrix can be viewed as an effective connectivity of the output projections of M1 to the muscles, during arm posture control. **D.** Illustration of decomposition of single trials into spatial linear and nonlinear components. *Left*, an example response function in blue in the 2D format (same as **Fig 2C**) but with mean firing rates drawn as residuals of spatial linear tuning-curve. Orange dots are randomly selected single-trials. *Top left*, magnifies one condition (*c*) showing the decomposition of a single trial (*t*) into the sum of: (i) mean spatial linear component, (ii) mean nonlinear component, (iii) single-trial noise fluctuation. *Top right*, example of the resulting purely nonlinear component with single-trial noise; note that there is no remaining linear component. *Bottom left*, same for the resulting purely spatial-linear component with single-trial noise.
(TIF)

S5 Fig 4 example response functions generated by the 2-parameter version of the neural network model following the same format as Fig 2. Examples were chosen to highlight the spatial-linearity and various forms of nonlinearity seen in Fig 2.
(TIF)

S1 Text Tuning is not the result of hand jitter. (PDF)

S2 Text Subpopulations for decoding EMG. (PDF)
