# Supplementary figures and images for: Tuning Curves for Arm Posture Control in Motor Cortex Are Consistent with Random Connectivity
Source: PLoS Comput Biol. 2016 May 25;12(5):e1004910. doi: 10.1371/journal.pcbi.1004910 (PMC4880440; doi:10.1371/journal.pcbi.1004910)

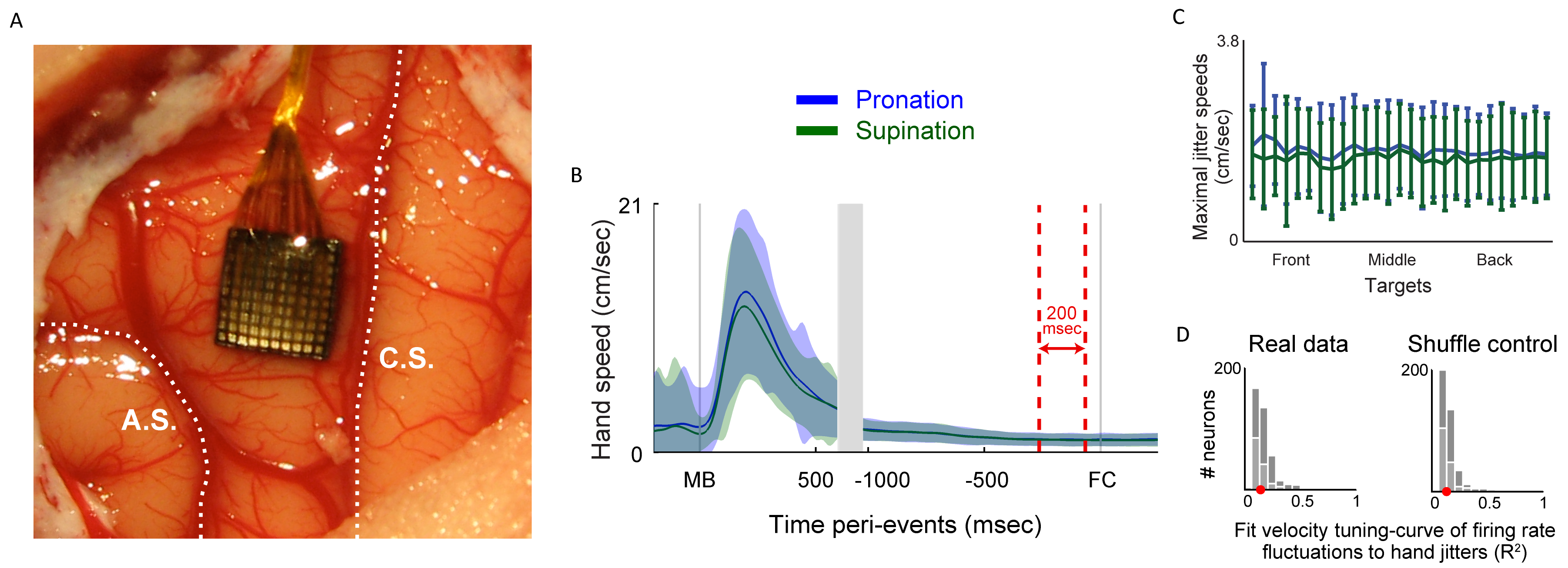

Supplement: S1 Fig — A. Chronic electrode array after insertion (for monkey BR; same location was used for monkey PK). C.S. = Central sulcus, A.S. = Arcuate sulcus. B. Mean ± S.D. of hand speed during the reaching movements to the target and the target-hold epochs, for each forearm posture. The 200 ms window analyzed in this study is denoted in red. Time is relative to task events; MB = Movement Beginning, and FC = Forearm rotation angle Cue (first sensory cue of next trial). Wide gray bar overlays the period where trials had random length intervals. Note that the hand speed is extremely low during target hold, yet not identically zero, because the hand was held freely in space. The larger variance before movement beginning is during the reaction time, and the larger variance during movement is due to averaging over reaches of different amplitudes. C. Mean ± S.D. of maximal hand jitter speed per trial, as a function of target position, and for each forearm posture. The hand jitter was uniform across targets (Multiple comparisons, using ANOVA, p > 0.01). D. Distribution of R2 values for fitting single trial firing rate fluctuations to hand jitter using the velocity tuning-curve, median = 0.12. Values with a significant fit (F-Test, p < 0.01) are in dark gray, while the rest are in light gray. Fitting to all tuned neurons (N = 411) and the optimal R2 for each neuron across all leads/lags (of firing rate to behavior) and forearm angles is presented. E. Same as D for control that shuffled single-trial firing rate fluctuations relative to their respective jitter movements, median = 0.11. (TIF) [file pcbi.1004910.s001.tif]

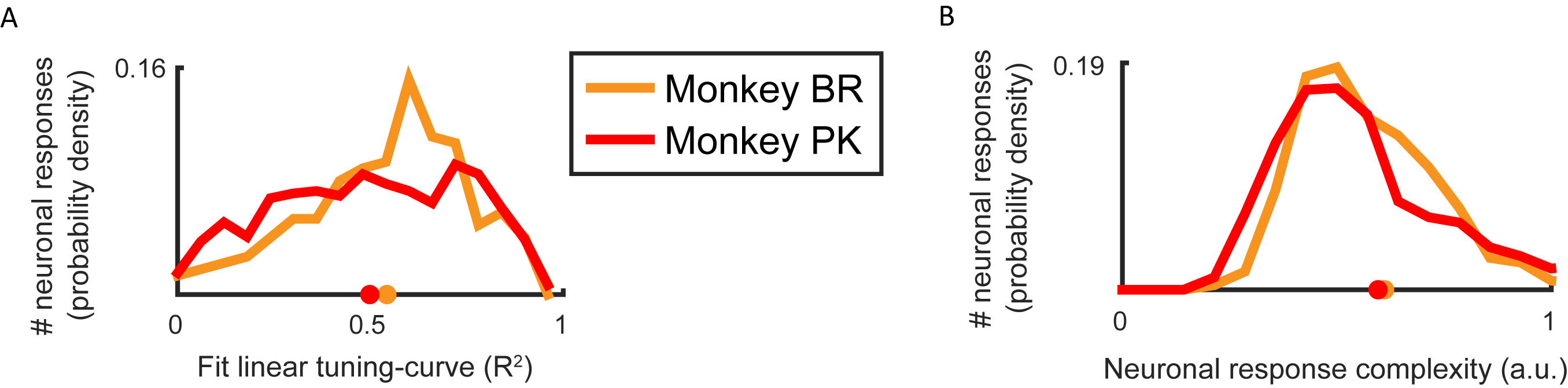

Supplement: S2 Fig — A. Distributions of R2 values for fit of linear tuning-curve for all tuned neurons (same as Fig 3B), separately from each monkey (means = 0.54 and 0.5, for monkeys BR and PK, respectively). B. Distributions of complexity measure of the (normalized) response functions (same as Fig 3D), separately for each monkey (means = 0.5 and 0.49, for monkeys BR and PK, respectively). (TIF) [file pcbi.1004910.s002.tif]

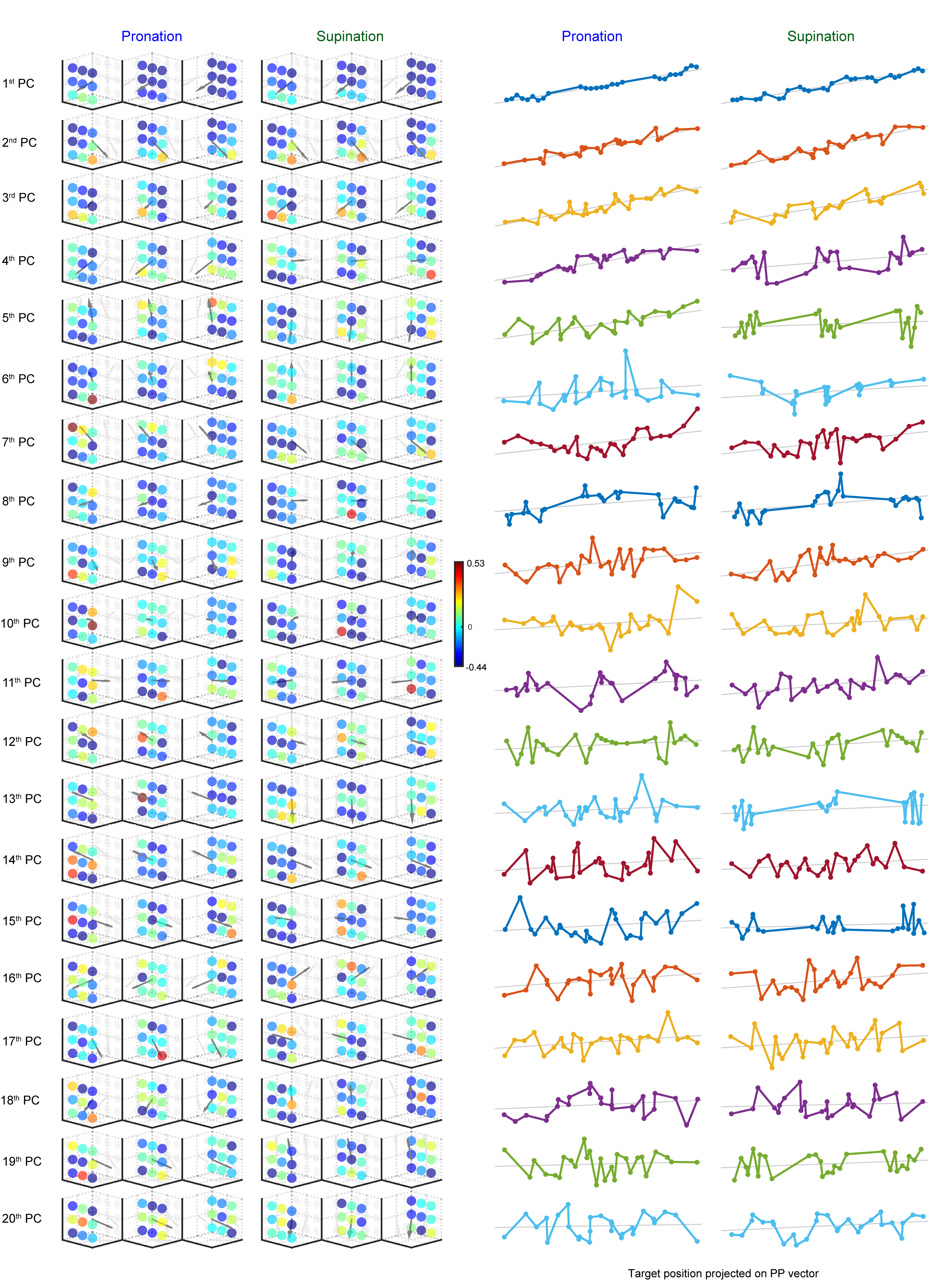

Supplement: S3 Fig — (TIF) [file pcbi.1004910.s003.tif]

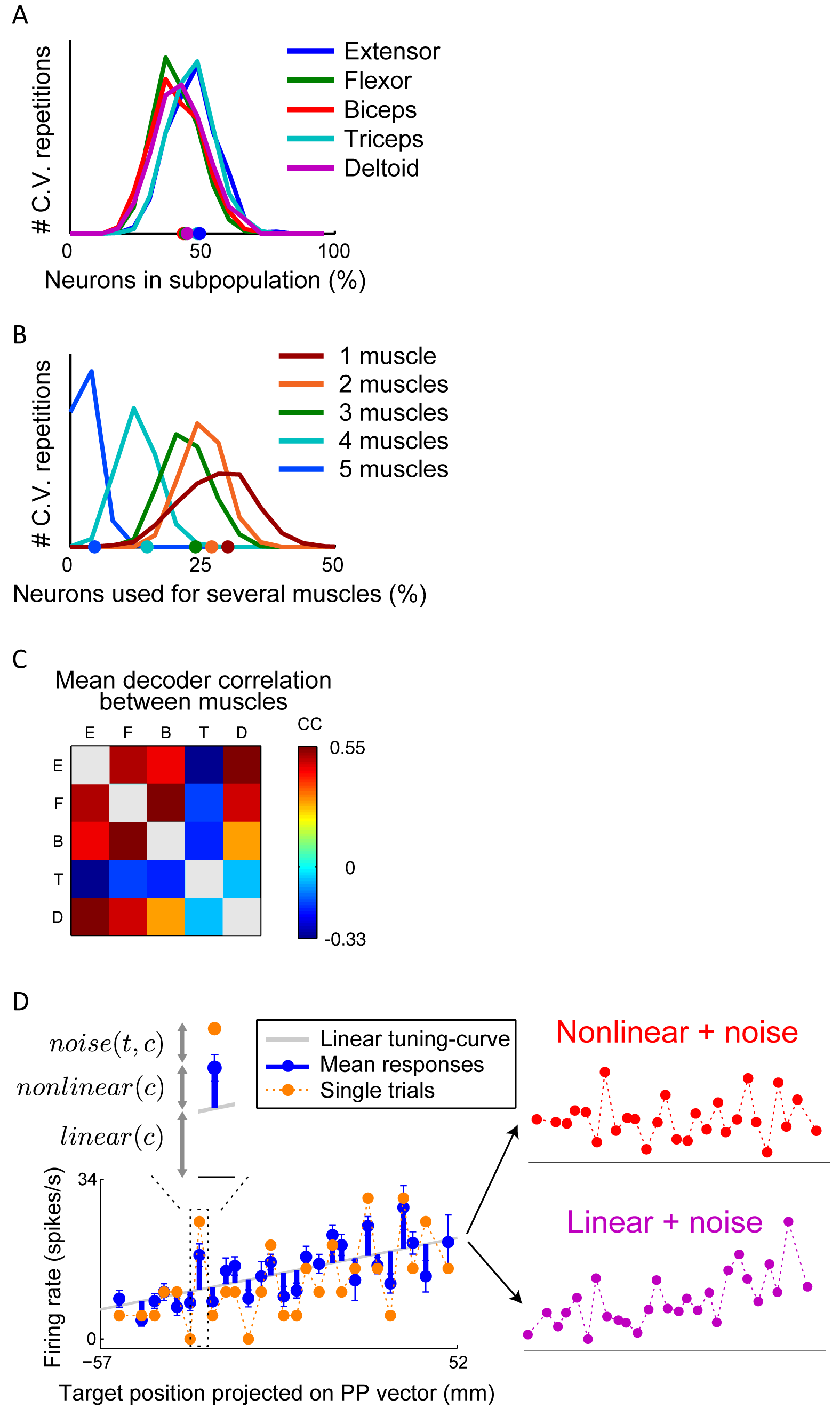

Supplement: S4 Fig — A. Percent of neurons selected for decoding each muscle, with distributions over cross-validation repetitions. Medians = 49%, 43%, 43%, 48%, 44%, for the forearm extensor, flexor, biceps, triceps, and deltoid, respectively (grand total median = 44%). B. Same as A., for percent of neurons selected for decoding any single muscle, any pair of muscles, etc., in each cross-validation repetition. Medians = 30% selected for any single, 27% for any pair, 24% for triples, 15% for quadruples, and 5% of neurons selected for decoding all 5 muscles. C. Matrix of mean correlations between decoders for each pair of muscles (only significant correlation coefficients were used, p < 0.01, Bonferroni corrected), averaged over cross-validation repetitions. This matrix can be viewed as an effective connectivity of the output projections of M1 to the muscles, during arm posture control. D. Illustration of decomposition of single trials into spatial linear and nonlinear components. Left, an example response function in blue in the 2D format (same as Fig 2C) but with mean firing rates drawn as residuals of spatial linear tuning-curve. Orange dots are randomly selected single-trials. Top left, magnifies one condition (c) showing the decomposition of a single trial (t) into the sum of: (i) mean spatial linear component, (ii) mean nonlinear component, (iii) single-trial noise fluctuation. Top right, example of the resulting purely nonlinear component with single-trial noise; note that there is no remaining linear component. Bottom left, same for the resulting purely spatial-linear component with single-trial noise. (TIF) [file pcbi.1004910.s004.tif]

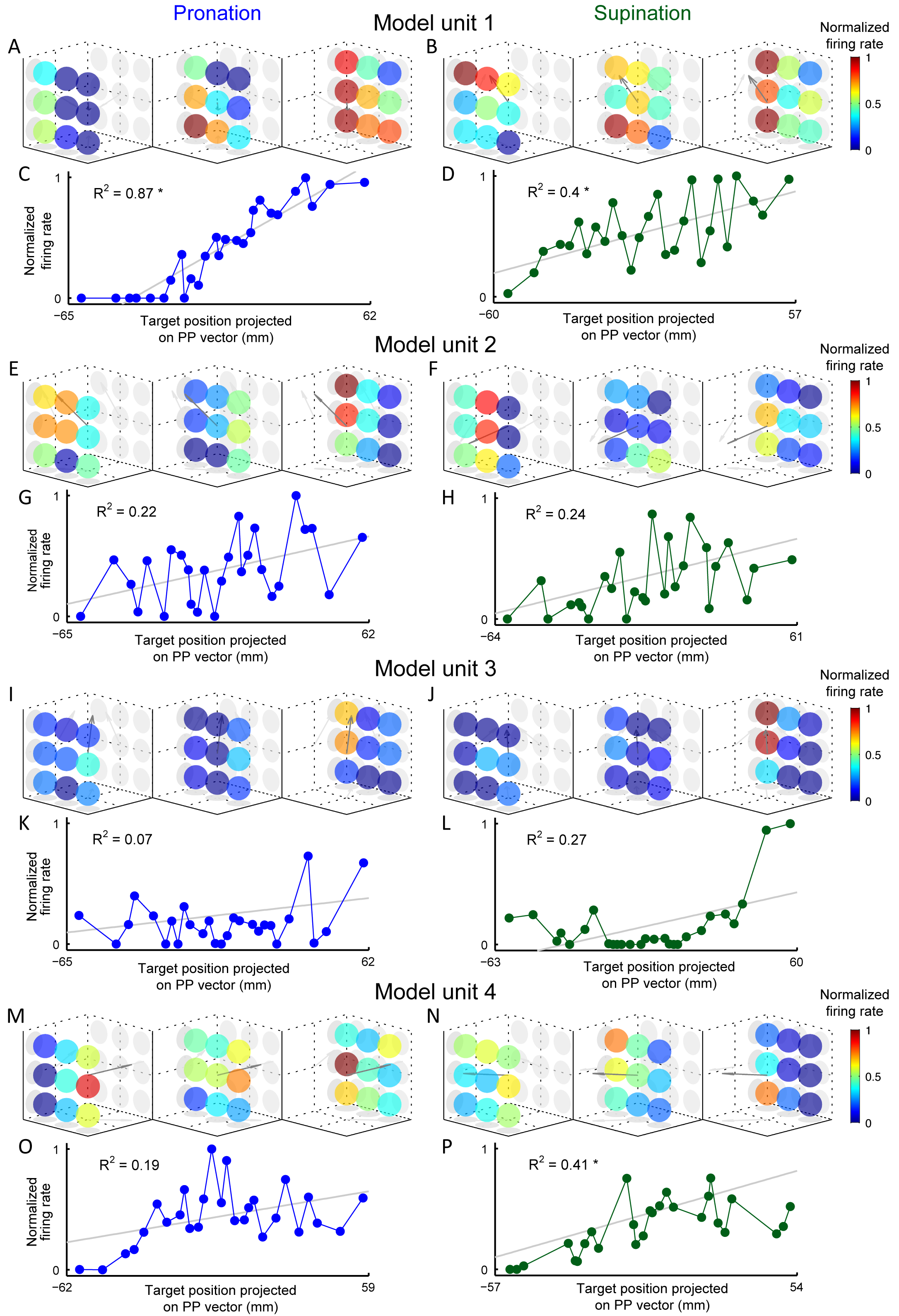

Supplement: S5 Fig — Examples were chosen to highlight the spatial-linearity and various forms of nonlinearity seen in Fig 2. (TIF) [file pcbi.1004910.s005.tif]
