# Supplementary material for: Tuning Curves for Arm Posture Control in Motor Cortex Are Consistent with Random Connectivity
Source: PLoS Comput Biol. 2016 May 25;12(5):e1004910. doi: 10.1371/journal.pcbi.1004910 (PMC4880440; doi:10.1371/journal.pcbi.1004910)
Supplement: S1 Text — (PDF) [file pcbi.1004910.s006.pdf]

Tuning is not the result of hand jitter

As the monkeys held their arms freely in space without the use of a manipulandum, their hands exhibited small jitter during the target hold epoch, with a mean speed of 1.2 ± S.D. 0.7 cm/s (for comparison, the average maximal hand speed during reaching was 14.5 ± S.D. 6.8 cm/s). Correlations between jitter velocity and target position could cause a movement-dependent signal to be included in the neuronal tunings we report.

We analyzed these minute movements (Methods) and found that the distributions of maximal jitter speed per trial (S1C Fig) were uniform across targets (multiple-comparisons, *ns*) suggesting they do not contribute to target tuning. Furthermore, when we repeated the tuning analysis using only trials with a maximal jitter speed below 2.3 cm/s (the $2/3^{rd}$ quintile of maximal jitter speeds per trial), the number of neurons significantly tuned did not change (other choices of the threshold produced similar results). These controls suggest that the tunings reflect correlations between firing rates and arm posture, and not hand jitter.

We also examined the relationship between hand jitter and fluctuations in firing rates from trial to trial, using the multiplicative velocity tuning model [1] (Methods) to characterize how single-trial firing rates were affected by hand jitter. The R^2 distribution for these fits was skewed towards zero (S1D Fig; median = 0.12), with values only marginally (but significantly, Wilcoxon rank-sum test, $p < 0.02$) larger than for a shuffle control (median = 0.11). The weakness of this correlation suggests that the postural tuning primarily reflects a feedforward motor intention rather than ongoing feedback control.

References

1. Moran DW, Schwartz AB. Motor cortical representation of speed and direction during reaching. The Journal of Neurophysiology. 1999;82(5):2676-92.
