# Supplementary material for: Tuning Curves for Arm Posture Control in Motor Cortex Are Consistent with Random Connectivity
Source: PLoS Comput Biol. 2016 May 25;12(5):e1004910. doi: 10.1371/journal.pcbi.1004910 (PMC4880440; doi:10.1371/journal.pcbi.1004910)
Supplement: S2 Text — (PDF) [file pcbi.1004910.s007.pdf]

Subpopulations for decoding EMG

Finding optimal subpopulations for decoding has the added benefit of providing information about how the signals that can drive different muscles are distributed across the M1 population. The distributions of subpopulation sizes, for each muscle separately, show the same overall shape with an overall mean of 45% of neurons selected for describing each EMG (S3A Fig). This is greater than the 20% expected if each subpopulation accounted for a single muscle. While these exact percentages depend on LASSO's meta-parameter (chosen via cross-validation and in the same manner for all cases; Methods), their relative sizes for the different muscles do not. Moreover, these subpopulations partially overlapped (neurons contributing to decoding more than one muscle), with medians of 30%, 27%, 24%, 15%, and 5% of neurons selected for decoding single muscles, pairs of muscles, triplets, 4 muscles, or all 5 muscles, respectively (S3B Fig). Finally, we calculated a matrix of the average correlation (over C.V. repetitions) between the decoders for each pair of muscles, which shows their similarity both in term of neurons selected and their decoding weight (S3C Fig; only significant correlation coefficients were used, $p < 0.01$, Bonferroni corrected). This matrix provides a map of the overlap in M1 subpopulations for decoding these muscles. For example, the decoders for the triceps are not correlated to those for the deltoid and anti-correlated with the decoders for the biceps, which is consistent with being an antagonistic muscle pair. In contrast, since in this task their role is primarily to maintain forearm rotation (the wrist was held in a neutral posture), the decoders for the forearm flexor and extensor are highly correlated. Taken together, these results show that not only is each muscle best predicted by only a subset of neurons, but that these subpopulations partially overlap, allowing components that are needed for more than one muscle to be shared.
